# Enhanced CO_2_ Electroreduction to Multi‐Carbon Products on Copper via Plasma Fluorination

**DOI:** 10.1002/advs.202309963

**Published:** 2024-03-27

**Authors:** Ziqian Zhou, Xiaosong Hu, Jiye Li, Haijiao Xie, Liaoyong Wen

**Affiliations:** ^1^ School of Materials Science and Engineering Zhejiang University Hangzhou 310027 China; ^2^ Research Center for Industries of the Future (RCIF) School of Engineering and Key Laboratory of 3D Micro/Nano Fabrication and Characterization of Zhejiang Province School of Engineering Westlake University Hangzhou 310024 China; ^3^ Hangzhou Yanqu Information Technology Co., Ltd Hangzhou 310003 China

**Keywords:** CO_2_ electroreduction, fluorinated copper catalysts, multi‐carbon products, plasma fluorination

## Abstract

The electroreduction of carbon dioxide (CO_2_) to multi‐carbon (C_2+_) compounds offers a viable approach for the up‐conversion of greenhouse gases into valuable fuels and feedstocks. Nevertheless, current industrial applications face limitations due to unsatisfactory conversion efficiency and high overpotential. Herein, a facile and scalable plasma fluorination method is reported. Concurrently, self‐evolution during CO_2_ electroreduction is employed to control the active sites of Cu catalysts. The copper catalyst modified with fluorine exhibits an impressive C_2+_ Faradaic efficiency (FE) of 81.8% at a low potential of −0.56 V (vs a reversible hydrogen electrode) in an alkaline flow cell. The presence of modified fluorine leads to the exposure and stabilization of high‐activity Cu^+^ species, enhancing the adsorption of *CO intermediates and the generation of *CHO, facilitating the subsequent dimerization. This results in a notably improved conversion efficiency of 13.1% and a significant reduction in the overpotential (≈100 mV) for the C_2+_ products. Furthermore, a superior C_2+_ FE of 81.6% at 250 mA cm^−2^, coupled with an energy efficiency of 31.0%, can be achieved in a two‐electrode membrane electrode assembly electrolyzer utilizing the fluorine‐modified copper catalyst. The strategy provides novel insights into the controllable electronic modification and surface reconstruction of electrocatalysts with practical potential.

## Graphical abstract

A facile and scalable plasma fluorination method is proposed to manufacture integrated gas diffusion electrodes of fluorine‐modified copper catalysts, which can precisely regulate the amount and depth of fluorine implantation on the copper to exhibit an impressive multi‐carbon products Faradaic efficiency of 81.8% at a low potential of −0.56 V in an alkaline flow cell.

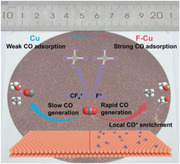

## 1 Introduction

Electrocatalytic CO_2_ reduction reaction (CO_2_RR) offers a promising solution to the energy and environmental crisis by transforming CO_2_ into valuable fuels or chemical feedstocks using renewable and clean electricity.^[^ ^1^ ^]^ The intricacy of CO_2_RR, which involves the transfer and coupling of multiple electrons and protons, leads to a broad distribution of products.^[^ ^2^ ^]^ Notably, the multi‐carbon (C_2+_) products, such as ethylene, ethanol, and propanol, with higher energy densities and economic value, take precedence over mono‐carbon (C_1_) products like carbon monoxide, formic acid, and the by‐product hydrogen.^[^ ^3^ ^]^ A primary challenge for C_2+_ products lies in carbon–carbon bond (C–C) coupling, stemming from the uncontrollable adsorption and combination of intermediates. At present, the selective catalysis of CO_2_ electroreduction for C_2+_ hydrocarbons predominantly focuses on copper‐based catalysts, attributed to their appropriate binding energy for *CO and *H intermediates.^[^ ^4^ ^]^ However, fierce competition from reactions involving low electron and proton transfer limits the highly selective conversion of C_2+_ products on copper catalysts. Consequently, multiple strategies, including electronic structure tuning,^[^ ^5^ ^]^ morphology engineering,^[^ ^6^ ^]^ tandem catalyst design,^[^ ^7^ ^]^ and surface molecule modification,^[^ ^8^ ^]^ have been reported to improve the selectivity and activity of Cu‐based catalysts for C_2+_ compounds.

In these significant studies, Cu^+^ species in copper‐based catalysts have been identified as the active sites for CO_2_RR to C_2+_ products, as evidenced by numerous operando spectroscopic tests and theoretical studies. However, Cu^+^ species suffer from thermodynamic instability during catalytic processes.^[^ ^9^ ^]^ Accordingly, heteroatoms such as sulfur, boron, and halogens (e.g., F, Cl, Br, and I) have been explored to stabilize the Cu^+^ active species and facilitate efficient C–C coupling.^[^ ^6^, ^10^ ^]^ Specifically, fluorine, being the most electronegative halogen element, is theoretically more conducive to the formation of active Cu^+^ species on Cu‐based catalysts. Moreover, Wang et al. reported that fluorine can strengthen water activation and hydrogenation of intermediates, leading to efficient hydrogen‐assisted C–C coupling.^[^ ^4b^ ^]^ Nevertheless, the halogen‐based copper catalysts are typically synthesized using wet chemistry methods, including hydrothermal, ice bath, and anodic oxidation.^[^ ^4^, ^10^, ^11^ ^]^ These wet chemistry methods require strict wet reaction conditions, showing poor controllability for the doping amount and depth, along with obvious environmental issues. Furthermore, these methods are limited to the modification of powder catalysts on traditional coated electrodes, lacking operability for integrated electrodes.

In this study, we propose a facile and scalable plasma fluorination method on Cu to create a cost‐effective F–Cu catalyst. The method enables precise control over the amount and depth of fluoride implantation on the Cu surface. When combined with a simple metal evaporation method, this approach allows for the preparation of a large‐scale integrated gas diffusion F–Cu catalyst electrode. The optimal F–Cu catalyst demonstrates a notable C_2+_ Faradaic efficiency (FE) of 81.8% at a low potential of −0.56 V versus a reversible hydrogen electrode (versus RHE) in a flow cell. In comparison to the reported Cu‐based catalysts modified by nonmetallic heteroatoms, the F–Cu catalyst prepared in this study displays remarkable C_2+_ selectivity at a lower overpotential. The in situ leaching of the fluorine in the F–Cu catalyst exposes more reactive sites in CO_2_RR. The adsorbed fluorine ions play a crucial role in generating and stabilizing Cu^+^ species, enhancing the adsorption and hydrogenation of *CO intermediates, and thereby promoting the conversion of C_2+_ products. This study introduces a highly controllable dry chemical fluorination method with potential applications in the synthesis of various functional materials.

## 2 Results and Discussion

**Figure 1:**
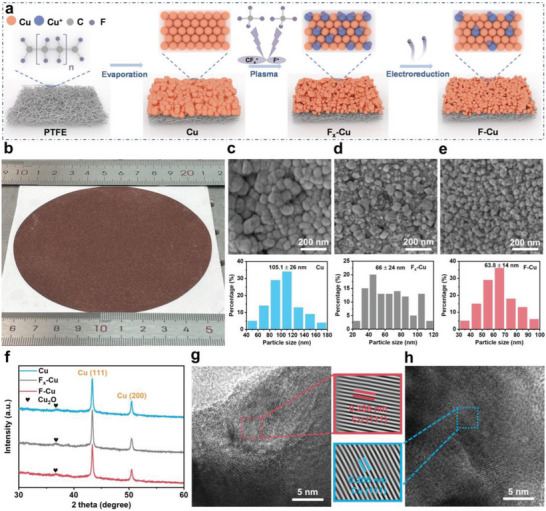
a) Schematic illustration of the preparation of the F–Cu catalyst. b) Photograph of F_x_‐Cu pre‐catalyst after plasma fluorination. SEM images and the corresponding size distributions of particles of c) Cu catalyst, d) F_x_‐Cu pre‐catalyst, and e) F–Cu catalyst. f) GI‐XRD patterns at an incidence angle of 0.15° with a theoretical penetration depth of 65 nm. HRTEM images of g) F–Cu catalyst and h) Cu catalyst.

The preparation process, encompassing plasma fluorination and in situ electroreduction, is illustrated in **Figure** **1**a. Initially, the Cu catalyst was fabricated via thermal evaporation on a gas diffusion layer. Subsequently, plasma fluorination was conducted in a CF_4_ atmosphere, wherein CF_4_ gas decomposes into CF_x_ and F active particles with high reactivity under the irradiation of high‐energy electrons. The F active particles then bombard the Cu surface, facilitating the formation of Cu─F bonds with Cu atoms and completing the fluorination process.^[^ ^12^ ^]^ Ultimately, the F–Cu catalyst was obtained through the electroreduction process, stabilizing the surface fluorine through in situ release of excess and unstable fluorine. This dry chemical method is facile and scalable, enabling the reliable preparation of large‐scale integrated gas diffusion electrodes utilizing the F–Cu catalyst (Figure 1b).

The subtle reconstruction of the surface structure during the preparation process was confirmed. In contrast to the dense particle arrangement in the Cu catalyst (Figure 1c; Figure S1, Supporting information), the high‐energy particles in the plasma caused the fragmentation of most surface Cu particles into smaller Cu nanoparticles (Figure 1d). Afterward, the in situ leaching of excess fluorine during electroreduction and structure reconstruction led to the dispersion of nanoparticles in the F–Cu catalyst (Figure 1e), enhancing the exposure of additional catalytic active sites. In addition, the surface roughness of the F–Cu catalyst (46.6 nm) also shows a substantial decrease compared to the Cu catalyst (71.4 nm) (Figure S2, Supporting information). It is worth noting that the reduction of catalyst particle size is unaffected by the power level of the plasma, exhibiting the reliability of the method (Figures S3 and S4, Supporting information).

The crystalline phase of the F–Cu catalyst during preparation was validated through grazing incident X‐ray diffraction (GI‐XRD, Figure 1f) and normal X‐ray diffraction (XRD, Figure S5, Supporting information), revealing no observed crystal phase change. The Cu (111) planes consistently dominate the catalyst components, with minimal Cu_2_O generation due to slight air oxidation. The high‐resolution transmission electron microscopy (HRTEM) images of the F–Cu catalyst (Figure 1g) and Cu catalyst (Figure 1h) reveal the identical interplanar spacing of 0.208 nm corresponding to the Cu (111) facet, reaffirming the consistency of the crystal phase.

In general, defects are readily generated during the plasma process^.[^ ^13^ ^]^ Electron paramagnetic resonance (EPR) and XRD enable the acquisition of crucial information regarding catalyst defect levels. Specifically, an increased EPR signal intensity and a decreased XRD peak intensity signify the augmented defect density.^[^ ^11^, ^14^ ^]^ Particularly, analyses of incidence‐angle dependent XRD (Figure S6, Supporting information) and EPR (Figure S7, Supporting information) reveal no substantial difference in defect density between the F–Cu catalyst and the pristine Cu catalyst. This observation may be attributed to the distinctive nature of plasma fluorination compared to conventional plasma processes or the instability of defects during the electrochemical reduction process. During plasma fluorination, chemisorption and bonding of the active F atoms predominantly occur, as opposed to the physical displacement of bulk atoms or their substitution by other atoms.

**Figure 2:**
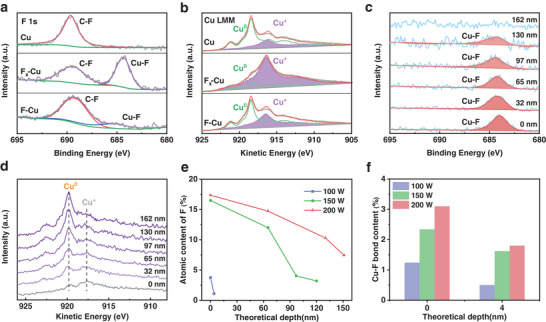
a) F 1s XPS spectra and b) Cu LMM Auger spectra of Cu catalyst, F_x_‐Cu pre‐catalyst, and F–Cu catalyst after slight surface etching with Ar^+^ beam. c) F 1s XPS spectra and d) Cu LMM Auger spectra of F_x_‐Cu‐200 W concerning different Ar^+^ beam etching depths. The variation of Cu–F species content with depth of e) F_x_‐Cu pre‐catalyst and f) F–Cu catalyst at different powers. The etching depths are estimated values based on theoretical parameters.

X‐ray photoelectron spectroscopy (XPS) was employed to verify the chemical state evolution on the surface of the F–Cu catalyst throughout. F 1s spectra (**Figure** **2**a) exhibit the existence of Cu─F bonding (685.1 eV) in both the F_x_‐Cu pre‐catalyst and F–Cu catalyst,^[^ ^4^, ^15^ ^]^ illustrating the successful chemical modification of copper by fluorine. The positive shift of Cu 2p peaks on the F–Cu catalyst also proves the fluorine modification (Figure S8, Supporting information). A notable decrease in the content of Cu–F bonding is observed in the F–Cu catalyst compared to the F_x_‐Cu pre‐catalyst, indicating the release of fluorine during the in situ electroreduction. Additionally, the peak at 689.7 eV is attributed to the C─F bonding originating from the PTFE substrate. Cu LMM Auger spectra in Figure 2b reveal the coexistence of Cu^0^ (918.5 eV) and Cu^+^ (916.4 eV) species on the catalysts.^[^ ^16^ ^]^ Following plasma fluorination, there is a significant increase in Cu^+^ content, accounting for ≈90%. After in situ electroreduction, the Cu^+^ content in the F–Cu catalyst decreases as the fluorine content diminishes, yet its Cu^+^ content (41.1%) remains higher than that of Cu catalyst (27.9%), as shown in Figure S9 (Supporting information).

XPS deep profiling, conducted through argon ion etching, provides additional insights into the correlation between plasma fluorination and Cu^+^ species. To ensure the result integrity, we directly used copper foil as the precursor to prevent any potential interference from fluorine in PTFE. As shown in Figure 2c, the fluorination intensity of plasma gradually decreases from the upper surface to the lower layer of the sample, corresponding to the relative decrease of the Cu^+^ species (Figure 2d). Moreover, the fluorination depth and the content of fluorine and Cu^+^ species enhance with the power increase from 100 W to 200 W (Figure 2e, Figures S10 and S11, Supporting information), which shows the controllability of plasma fluorination. Remarkably, after electroreduction in 1 M KOH, a substantial decrease is observed in the levels of fluorine and Cu^+^ species, primarily situated within the surface region of several nanometers, due to the in situ release of fluorine in the electrochemical environment (Figures S12 and S13, Supporting information). However, the subsequent enrichment of fluorine on the surface remains positively correlated with the applied power (Figure 2f).

**Figure 3:**
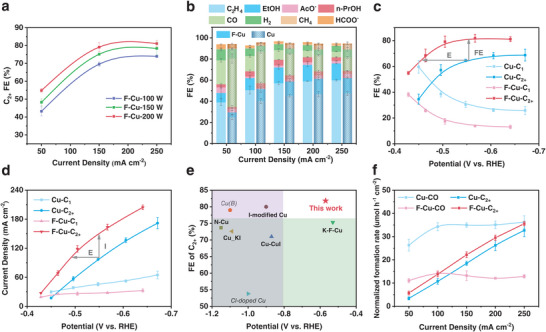
a) C_2+_ FE values of F–Cu catalysts with different power. b) FE values of different products on F–Cu and Cu catalysts. c) FE values and d) partial current densities of C_2+_ and C_1_ on Cu and F–Cu catalysts. e) Comparison of electrochemical CO_2_RR performance on F–Cu catalyst with reported Cu‐based catalysts modified by nonmetallic heteroatoms. f) Normalized formation rates by ECSA of CO and C_2+_ products for Cu and F–Cu catalysts. Error bars represent the standard deviation of measurements based on three independent samples.

Electrocatalytic CO_2_RR performances were evaluated in a flow cell with 1 M KOH electrolyte under ambient conditions. Potentials in the flow cell system were transformed to the reversible hydrogen electrode scale with iR correction. **Figure** **3**a presents the CO_2_RR performance of the F–Cu catalyst at different fluorination powers (100 to 200 W), showing an overall improvement in C_2+_ FE with increasing power (Figure S14, Supporting information). Therefore, the F–Cu catalyst at 200 W was used as our optimal sample for subsequent characterization.

We measured linear sweep voltammetry (LSV) for the F–Cu catalyst and Cu catalyst under both CO_2_ and argon atmospheres. The F–Cu catalyst exhibited higher current density under the CO_2_ atmosphere compared to the Cu catalyst across the entire test potential range (Figure S15, Supporting information), while both catalysts delivered similar current densities under the argon atmosphere. It means that the F–Cu catalyst possesses higher CO_2_RR activity. Concerning the products, the main C_2+_ product is ethylene, along with ethanol and small amounts of acetic acid and n‐propanol (Figure S16, Supporting information). Thereinto, the ethylene (C_2_H_4_) FE values increase with decreasing CO FE in the current density range of 50 to 250 mA cm^−2^ for both Cu and F–Cu catalysts. The F–Cu catalyst notably promoted the selectivity of C_2_H_4_ (Figure 3b). At a current density of 250 mA cm^−2^, the C_2_H_4_ FE and CO FE of the F–Cu catalyst were 59.9% and 5.3%, respectively, compared to 47.4% and 12.6% for the Cu catalyst. This result suggests that the Cu catalyst, after fluorination, can expedite the conversion of C_1_ products to C_2+_ products. Most importantly, the upper FE of C_2+_ was increased by 13.1% for the F–Cu catalyst, accompanied by a significant reduction in overpotential (≈100 mV, Figure 3c). Meanwhile, the C_2+_ partial current densities of the F–Cu are almost 1.5 times higher than those of the Cu catalyst at various overpotentials (Figure 3d). Notably, our F–Cu catalyst demonstrates superior selectivity for C_2+_ products, with an impressive FE of 81.8% and a low potential at −0.56 V versus RHE. This performance outperforms Cu‐based catalysts modified with nonmetallic heteroatoms using wet‐chemical approaches, as reported in the literature (Figure 3e; Figure S17, Supporting information).

Besides, the electrocatalytic stability of the F–Cu catalyst was measured at a current density of 200 mA cm^−2^, revealing a noteworthy stability over 12 h (Figure S18a, Supporting information). Previous studies have identified flooding and carbonate accumulation at the cathode as hindrances to the diffusion and adsorption of CO_2_, resulting in reduced activity and selectivity. These factors represent significant contributors to the poor stability of CO_2_RR in flow cells.^[^ ^17^ ^]^ Through the implementation of an electrolyte update strategy aimed at eliminating temporary carbonate accumulation and flooding, we successfully restored both selectivity and activity (Figure S18, Supporting information), thereby highlighting the intrinsic electrocatalytic stability of the F–Cu catalyst. Moreover, the F–Cu catalyst maintained consistent morphology and Cu (111) facet (Figure S19, Supporting information), as well as stable fluorine modification and Cu^+^ content (Figure S20, Supporting information) after a prolonged CO_2_RR process. These results demonstrate the reliability of the F–Cu catalyst during long‐term electrocatalytic processes.

Next, we explored the factors contributing to the performance enhancement of the F–Cu catalyst. On the one hand, given that plasma fluorination induced no alterations in crystal structure and defects (Figure 1g,h; Figures S6 and S7, Supporting information), the changes in particle size and surface roughness resulting from structural reconstruction may account for the observed performance improvement.^[^ ^18^ ^]^ The electrochemically active surface area (ECSA), typically corresponding to the number of active sites, was calculated from the measured double‐layer capacitance.^[^ ^19^ ^]^ The ECSA of the F–Cu catalyst (19.1 cm^−2^) surpasses that of the Cu catalyst (16.2 cm^−2^), potentially explaining the increase in current density (Figures S15, Supporting information; Figure 3d). Moreover, since the ECSA and particle size of F–Cu catalysts at different powers exhibit similarity (Figure s4; Figure S21 and S22, Supporting information), the heightened content of surface fluorine with increasing fluorination power (Figure 2f) likely contributes to the improvement in the C_2+_ products (Figure 3a). The normalized formation rates by ECSA of CO and C_2+_ products further prove that fluorine modification enhances the intrinsic properties of C_2+_ production on the F–Cu catalyst (Figure 3f).

On the other hand, we employed the electrochemical impedance spectra (EIS) to investigate the reactive kinetics of the electrode interface (Figure S23, Supporting information). The charge‐transfer resistance (R_ct_) of the F–Cu catalyst measured 27.23 Ω cm^−2^, less than that of the Cu catalyst (60.47 Ω cm^−2^) at −0.2 V versus RHE. The R_ct_ of the F–Cu catalyst also decreases with the increase in fluorination power. The reduced R_ct_ manifests the faster catalytic kinetics of the F–Cu catalyst, including more favorable electron transfer and stronger ion adsorption capacity during the CO_2_RR process, which may be the reason for reduced overpotentials at the same current densities in Figure 3c,d. Furthermore, considering that the hydrophilicity of the catalyst is a key factor for reactivity and selectivity. We conducted contact angle tests using water solutions to compare the hydrophilicity of the samples. The results indicate that there were no significant differences in hydrophilicity among the samples (Figure S24, Supporting information), suggesting that fluorine modification did not enhance performance by altering the hydrophilicity. The original Cu catalyst, based on the PTFE substrate, already exhibited decent hydrophobicity due to the inherent properties of PTFE. Fluorine modification did not significantly enhance the hydrophobicity, especially evident in the sample treated with 150 W, where performance significantly improved without a corresponding increase in hydrophobicity. This observation strongly supports the conclusion.

**Figure 4:**
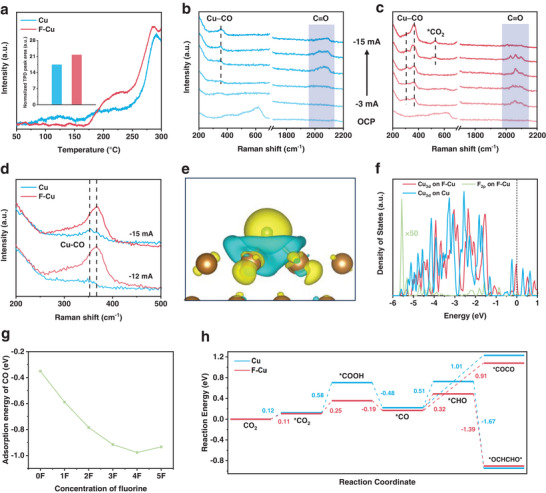
a) CO‐TPD profiles. In situ Raman spectra of the b) Cu and c) F–Cu catalysts under different current densities. OCP, open‐circuit potential. d) Comparison of in situ Raman spectra of Cu and F–Cu catalysts in the range of 200–500 cm^−1^. e) Charge density difference of F–Cu catalyst. f) Projected density of states (PDOS) of 3d orbitals of Cu atom, 2p orbitals of F atom on F–Cu and Cu catalyst. g) CO adsorption energy on F–Cu catalyst with different numbers of fluorine atoms. 0F, 1F, 2F, 3F, 4F, and 5F refer to Cu catalyst and fluorine‐modified Cu catalysts with concentrations of 0/16, 1/16, 1/8, 3/16, 1/4, and 5/16 monolayer, respectively. h) A reaction energy diagram of CO_2_RR on Cu (111) and F–Cu (111) surfaces.

To delve deeper into the thermodynamic impact of fluorine modification, we conducted a comprehensive investigation involving in situ characterization and theoretical calculations. The adsorption capability of catalysts for reactants and intermediates serves as an intuitive reflection of their intrinsic catalytic activity. Illustrated in Figure S25 (Supporting information), the CO_2_ physisorption capacity of the F–Cu catalyst (21.3 cm^3^ g^−1^) surpasses that of the Cu catalyst (17.6 cm^3^ g^−1^), enhancing the availability of reactant molecules around active sites and thereby conferring an advantage to CO_2_RR over competing processes. Beyond the adsorption quantity, temperature‐programmed desorption (TPD) provides insights into the adsorption strength of different catalysts for reactants or intermediates. Only the Cu catalyst exhibits prominent desorption peaks below 150 °C, indicating weak CO adsorption (**Figure** **4**a). In the high‐temperature region (>200 °C), the desorption peaks on the F–Cu catalyst appear at higher temperatures, demonstrating that the F–Cu catalyst predominantly exhibits strong adsorption of CO, with stronger adsorption strength compared to the Cu catalyst. Additionally, the overall desorption peak area for the F–Cu catalyst is greater than that of the Cu catalyst, indicating a stronger overall CO adsorption capacity and a greater number of adsorption sites (The inset in Figure 4a). This result substantiated the stronger CO chemisorption on the F–Cu catalyst, suggesting the potential enhancement of adsorption to *CO intermediates.^[^ ^10^, ^20^ ^]^

To gain genuine insights into the key *CO intermediate for C_2+_ pathways on the catalyst, we employed in situ Raman spectroscopy to monitor the CO_2_RR process, as shown in Figures 4b,c. At open circuit potential (OCP), peaks in the range of 330 to 675 cm^−1^ correspond to copper oxides, resulting from surface oxidation of Cu in the solution, which subsequently disappears at the reduction potentials. Notably, interactions between the catalytic interface and the *CO intermediate were observed, with characteristic peaks at ≈312 cm^−1^ and ≈365 cm^−1^ associated with the Cu–CO frustrated rotation and stretch, respectively.^[^ ^21^ ^]^ Additionally, the band within the range of 1900–2130 cm^−1^ can be ascribed to the C≡O stretch. These bands corresponding to surface‐absorbed *CO appeared at a lower current density on the F–Cu catalyst (−3 mA cm^−2^) compared to the Cu catalyst (−9 mA cm^−2^), indicating a smaller potential for CO production on the F–Cu catalyst. Furthermore, we observed a significant blue shift of Cu–CO on the F–Cu catalyst relative to the Cu catalyst (Figure 4d), indicating the stronger CO binding on the F–Cu catalyst than on the Cu catalyst.^[^ ^22^ ^]^ These results suggest that the F–Cu catalyst is more favorable for the generation and adsorption of CO intermediates. The combination of F–Cu catalysts with a reduced CO FE and an increased C_2+_ FE compared to Cu catalysts demonstrates that the enhanced adsorption of *CO promotes the conversion of C_2+_ products to some extent.^[^ ^23^ ^]^

Theoretical calculations using first‐principles density functional theory (DFT) were performed to further elucidate the mechanism underlying the promotion of the C_2+_ product on the F–Cu catalyst. To determine the type of surface fluorine adsorption, we calculated the adsorption energies at different adsorption sites, including top, bridge, and hollow sites. The results indicate that the hollow site is more favorable compared to the top and bridge sites, with the lowest adsorption energy (Figure S26, Supporting information). We conducted the charge density differences and projected density of states (PDOS) to investigate the difference in electronic structure between F–Cu and Cu catalysts. The charge density difference of the F–Cu catalyst illustrates the electron accumulation around the fluorine atom due to its strong electronegativity (Figure 4e), consistent with the observed increase in Cu^+^ content. Additionally, the modification of fluorine significantly affects the electron density near the Fermi energy level of Cu, resulting in a closer alignment of the d‐band center on F–Cu with the Fermi energy level (Figure 4f), thereby enhancing the adsorption of *CO intermediates.^[^ ^24^ ^]^

Furthermore, the calculations reveal that the introduction of fluorine indeed enhances the CO adsorption capability on the copper surface. The results indicate that with an increase in the number of fluorine atoms, the adsorption strength of CO on the F–Cu catalyst increases and tends toward a stable adsorption strength (Figure 4g; Figure S27, Supporting information). This finding is consistent with our experimental results, where an increase in fluorine content enhances CO adsorption, leading to reduced CO evolution and consequently improving the FE of C_2+_. This inherent improvement in CO adsorption aligns with the results obtained from CO‐TPD and in situ Raman. Through XPS data analysis, we calculated the relative atomic ratio of F to Cu in F–Cu‐200 W to be ≈6.4% (F/Cu atomic ratio) using the relative sensitivity factor method. Therefore, we conducted comparative studies of reaction energy using the fluorine‐modified copper (1/16 monolayer), aiming to better reflect the actual proportion of the catalyst.

Through the optimization of intermediate adsorption structures (Figures S28–S30, Supporting information) and the calculation of corresponding Gibbs free energy, we obtained the reaction energy pathways of CO_2_RR over the electrocatalyst interfaces. This comprehensive approach allows for an in‐depth understanding of product selectivity on both Cu and F–Cu catalysts (Figure 4h), enabling a detailed exploration of the reaction mechanism and the impact of fluorine. Crucially, the F–Cu catalyst promotes the formation of *CO intermediates through the favorable formation of *COOH, potentially attributed to the robust fluorine‐hydrogen interaction.^[^ ^4b^ ^]^ The accelerated production of *CO significantly improves the conversion efficiency of CO_2_, providing an ample supply of available intermediates for subsequent C–C coupling.^[^ ^25^ ^]^ Furthermore, the strong fluorine–hydrogen interaction also facilitates the hydrogenation of *CO to form *CHO intermediates on the F–Cu surface, with a reaction energy of 0.32 e V compared to 0.51 eV for Cu surface, followed by energy release to achieve dimerization into *OCHCHO*. Additionally, for the dimerization process with a high energy barrier from *CO to *COCO, the reaction energy on the F–Cu surface (0.91 eV) is also lower than that on the Cu surface (1.01 eV). The decreased reaction energy demonstrates the crucial role of fluorine modification in promoting *CO formation and C–C coupling. These results elucidate the intrinsic advantage of the F–Cu catalyst in generating multi‐carbon products, correlating with reduced overpotential and increased selectivity of C_2+_ conversion.

**Figure 5:**
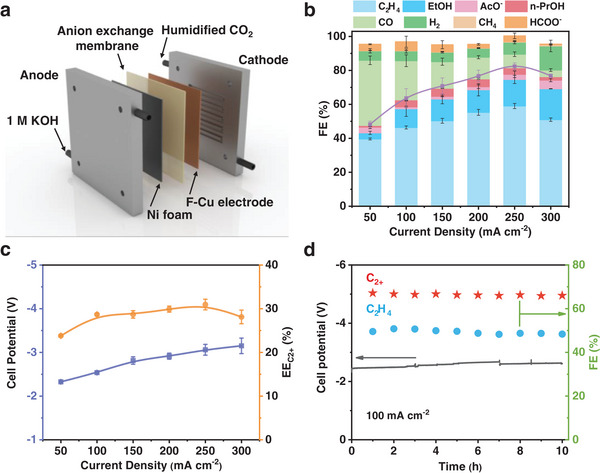
a) Schematic illustration of the MEA system. b) FE values of different products, c) cell potential and EE_C2+_, and d) electrochemical stability test throughout 10 h at the total current density of 100 mA cm^−2^ for F–Cu catalyst in a 1 cm^−2^ MEA electrolyzer. Error bars represent the standard deviation of three independent measurements.

To address electrode flooding issues and improve energy efficiency, we evaluated the CO_2_RR performance in the MEA system (**Figure** **5**a). The LSV curve of the F–Cu catalyst in the MEA system is shown in Figure S31 (Supporting information). It is evident that the FEs of the products at various current densities resemble those observed in the three‐electrode flow cell. Specifically, the C_2_H_4_ FE increases with rising current density, reaching a maximum of 55%, corresponding to a C_2+_ FE of 81.6% at 250 mA cm^−2^ (Figure 5b). Notably, the full‐cell EE without ohmic loss compensation for C_2+_ products reaches 31.0% at 250 mA cm^−2^ (Figure 5c). This value can be further enhanced by reducing resistance and employing efficient anodes, bringing it closer to practical industrial applications. Additionally, the F–Cu catalyst displayed excellent stability in the MEA system at 100 mA cm^−2^, with FEs for C_2_H_4_ and C_2+_ products remaining above 95% after 10 h of continuous electrolysis (Figure 5d). The marginal decrease in FEs can be attributed to the salt‐out effect on the electrode, a common challenge in alkaline CO_2_RR electrolysis.

## 3 Conclusion

This study reports a fluorinated Cu catalyst prepared via a facile and scalable plasma fluorination method, followed by in situ electroreduction. The introduction and in situ leaching of fluorine contributes to the stabilization and exposure of the active Cu^+^ species. Additionally, the extremely strong electronegativity of fluorine generates intense fluorine‐copper and fluorine‐hydrogen interactions, inherently enhancing the adsorption of crucial intermediate species *CO and the generation of *CHO, thereby facilitating C–C coupling. In comparison to the Cu catalyst, the F–Cu catalyst exhibits a substantial enhancement in C_2+_ FE (13.1%) with a lower overpotential (decreased by 100 mV). As a result, the F–Cu catalyst achieves a high C_2+_ FE of 81.8% (at −0.56 V vs RHE) in a three‐electrode flow cell and presents an 81.6% C_2+_ FE at 250 mA cm^−2^ in a two‐electrode MEA system, with an EE of 31.0%. Our work proposes an appealing strategy employing plasma fluorination to realize efficient and controllable fluorine modification on Cu catalysts, leading to highly selective electrosynthesis of C_2+_ products. This strategy exhibits promising potential for scalable non‐metallic doping on catalysts with fewer environmental concerns and improved performance.

## Conflict of Interest

The authors declare no conflict of interest.

## Supporting information

Supporting Information

## Data Availability

The data that support the findings of this study are available from the corresponding author upon reasonable request.
