# Supplementary material for: Enhanced CO2 Electroreduction to Multi‐Carbon Products on Copper via Plasma Fluorination
Source: Adv Sci (Weinh). 2024 Mar 27;11(22):2309963. doi: 10.1002/advs.202309963 (PMC11165481; doi:10.1002/advs.202309963)
Supplement: Supplementary file 1 — Supporting Information [file ADVS-11-2309963-s001.pdf]

ADVANCED SCIENCE
Open Access

Supporting Information

for *Adv. Sci.*, DOI 10.1002/advs.202309963

Enhanced CO_2 Electroreduction to Multi-Carbon Products on Copper via Plasma Fluorination

Ziqian Zhou, Xiaosong Hu, Jiye Li, Haijiao Xie and Liaoyong Wen**

Supporting Information

Enhanced CO_2 Electroreduction to Multi-carbon Products on Copper via Plasma Fluoridation

Ziqian Zhou, Xiaosong Hu, Jiye Li, Haijiao Xie, Liaoyong Wen**

Ziqian Zhou, Jiye Li

School of Materials Science and Engineering, Zhejiang University, Hangzhou 310027, China

Ziqian Zhou, Xiaosong Hu, Jiye Li, Liaoyong Wen

Research Center for Industries of the Future (RCIF), School of Engineering and Key Laboratory of 3D Micro/Nano Fabrication and Characterization of Zhejiang Province, School of Engineering, Westlake University, Hangzhou 310024, China

Haijiao Xie

Hangzhou Yanqu Information Technology Co., Ltd, Hangzhou 310003, China

E-mail: *wenliaoyong@westlake.edu.cn; xiaosong_hu@mail.nankai.edu.cn*

Methods

Materials

PTFE was purchased from Guangzhou Cleverflon new materials technology company. The anion-exchange membrane including Fumasep FAB-PK-130 and Sustainion® X37-50 were purchased from SCI Materials Hub and were activated in 1 M KOH before use. Deionized (DI) water with a resistivity of 18.2 MΩ·cm was obtained by the Milli-Q system (Millipore, Billerica, MA). Potassium hydroxide (KOH, AR, 99.0%) and potassium bicarbonate ($KHCO_3$, 99.5%) were purchased from Sinopharm Chemical Reagent Co., Ltd. Deuterated heavy water (D_2O), 2-methyl sulfoxide (DMSO, 99.7%), methanol (CH_3OH, 99.9%), ethanol (C_2H_5OH, 99.5%), isopropanol (C_3H_8O, 99.5%), formic acid (HCOOH, 99.0%), and acetic acid (CH_3COOH, 99.8%) were purchased from Shanghai Adamas Reagent Co., Ltd. All chemicals were used as it was without further treatment.

Fabrication of Cu catalyst

The polytetrafluoroethylene (PTFE) with a pore size of 1 μm was used as the substrate and gas diffusion layer. Approximately 500 nm Cu were evaporated on the substrate in ~10^{-4} Pa at 0.4 Å/sec.

Fabrication of F-Cu catalyst

The Cu catalyst was treated by CF_4 plasma (Inductively Coupled Plasma etching system from Leuven, ICP) for 1 minute with power of 200 W and 50 W RF bias at 5 mTorr and 20 °C (F_x-Cu). Then, the F_x-Cu pre-catalyst was reduced in 1 M KOH solution at −0.8 V versus RHE for 10 min to fabricate the F-Cu catalyst. The F-Cu catalyst under different powers can also be prepared, such as F-Cu-100 W and F-Cu-150 W by changing the plasma power.

Characterization

Phase components of the as-prepared catalysts were tested by X-ray diffractometer (XRD, Germany Bruker, D8 Advance) with Cu Kα radiation (λ = 0.15418 nm). Morphologies of the catalysts were confirmed by a field emission scanning electron microscope (FESEM, Hitachi High-Technologies Corporation, Regulus 8230) and a transmission electron microscope (TEM, Thermo Fisher Scientific, Talos F200X G2) equipped with energy-dispersive X-ray detectors (Oxford, Ultim EXTREME). X-ray photoelectron spectroscopic (XPS, Thermo Fisher Scientific, ESCALAB Xi+) was employed to detect the surface chemical state of samples. Electron paramagnetic resonance (EPR) measurements were carried out on a CIQTEK EPR200M spectrometer and microwave frequency = 9.40 GHz at room temperature. CO_2 sorption isotherms were measured using Micromeritics 3FLEX at 25 °C. TPD measurements were performed using Microtrac BELCat II.

Electrochemical measurements

The CO_2RR in the flow cell (Gaoss Union, 1 cm^2) separated by an anion-exchange membrane (FAA-3-PK-130, Fumasep) was controlled by a CHI 660e electrochemical workstation. The as-made catalysts were directly employed as the working electrode with Ag/AgCl electrode (reference electrode, 3 M KCl) and nickel foam (counter electrode). All the potentials were converted to values with reference to

the RHE using: E (RHE) = E (Ag/AgCl) + 0.21 V + 0.059 V × pH + *i*R, with 85% compensation. 50 mL 1 M KOH was used as catholyte and anolyte. The electrolytes in the cathode and anode were circulated by two pumps at the rate of 5 ml min^{-1} and 90 ml min^{-1}, respectively. Meanwhile, CO_2 gas was continuously supplied to the gas chamber of the cathode at the rate of 30 ml min^{-1}. The performance of the cathodes was evaluated by performing constant-current electrolysis. Gas and liquid products were analyzed using a gas chromatograph (GC, Shimadzu, GC-2014) equipped with thermal conductivity and flame ionization detectors and a nuclear magnetic resonance (NMR) spectrometer (Bruker BioSpin, AVANCE NEO) by taking dimethylsulfoxide (DMSO) as an internal standard, respectively.

For the electrochemical CO_2RR test in an MEA electrolyzer, a commercial MEA electrolyzer (Gaoss Union, 1 cm^2) was used. The F-Cu, an anion-exchange membrane (Sustainion® X37-50) and nickel foam were compressed to form MEA. 1.0 M KOH solution was served as the anolyte with a flow rate of 90 ml min^{-1}. The flow rate of cathodic CO_2 gas was kept at 30 sccm and flowed through a homemade humidifier (deionized water, room temperature) before the MEA. The electrolysis of the MEA electrolyzer was performed on a CHI 1140c electrochemical workstation.

The Faradaic efficiencies (FEs) were calculated on the basic of the following equation:

$$\mathrm{FE} = \frac{Q_x}{Q_{total}} = \frac{n_x N_x F}{Q_{total}}$$

where Q_x and Q_{total} were the charge passed into product x and passed charge (C) during CO_2RR, n_x represents the electron transfer number of product x, N_x was the product amount (mol) of x measured by GC or NMR and F was the Faraday constant (96485 C mol^{-1}).

The energy efficiencies (EEs) were calculated on the basic of the following equation:

$$\mathrm{EE} = \frac{1.23 - {E_x}^0}{E_{applied}} \times FE_x$$

where $E_{applied}$ represents the potential applied during the CO_2RR, E_x^0 is the

thermodynamic potential (vs. RHE) for the product x, such as 0.08 V for ethylene formation, 0.09 V for ethanol formation, 0.1 V for propanol formation and 0.11 V for acetic acid formation.[1]

In-situ Raman measurements

Raman spectroscopy was carried out in a custom-built flow cell using a Raman spectrometer (WITec Alpha 300R). A 785-nm laser was used with a laser power of 3 mW, and signals were recorded using a 5-s integration and by averaging two scans. A 10× objective lens was used for focusing and collecting the incident and scattered laser light. The electrochemical CO_2 reduction was performed at different currents from 0 to −15 mA cm^{-2} in 1 M $KHCO_3$.

Computational Methods

All calculations are performed in the framework of the density functional theory with the projector augmented plane-wave method, as implemented in the Vienna ab initio simulation package.[2] The generalized gradient approximation proposed by Perdew, Burke, and Ernzerhof is selected for the exchange-correlation potential.[3] The long-range van der Waals interaction is described by the DFT-D3 approach.[4] The cut-off energy for the plane wave is set to 450 eV. The energy criterion is set to 10^{-5} eV in the iterative solution of the Kohn-Sham equation. A large supercell (~10 Å× 10Å×21 Å) was adopted to simulate the Cu(111) and F-Cu(111) slab. An F atom was anchored on the surface through F-Cu bonds at the hollow site. A vacuum layer of 15 Å is added perpendicular to the sheet to avoid artificial interaction between periodic images. All the structures are relaxed until the residual forces on the atoms have declined to less than 0.03 eV/Å. During geometry relaxation, the bottom two layers were relaxed. For the reaction free energy calculations, a computational hydrogen electrode model was employed.[5] Gibbs free energies (G) were estimated as $G = E_{DFT} + G_{ZPE} + \Delta TS$. Here, E_{DFT} is the electronic energy computed from DFT calculations. The zero-point energy (G_{ZPE}) and thermal entropy (ΔTS) were evaluated using the VASPKIT code.[6]

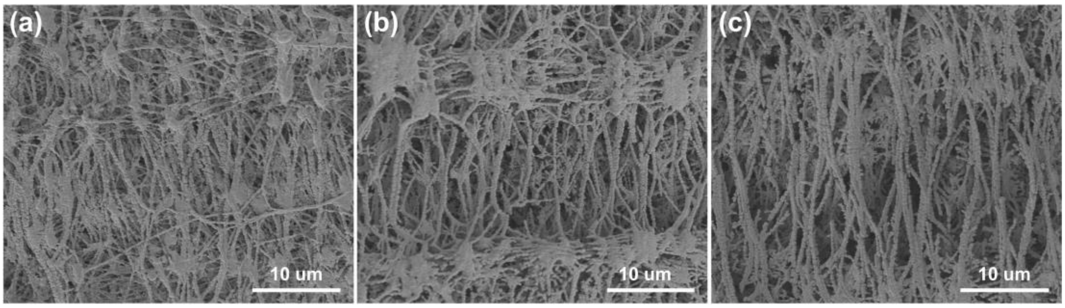

Figure S1. SEM images of (a) Cu catalyst, (b) F_x-Cu pre-catalyst, and (c) F-Cu catalyst.

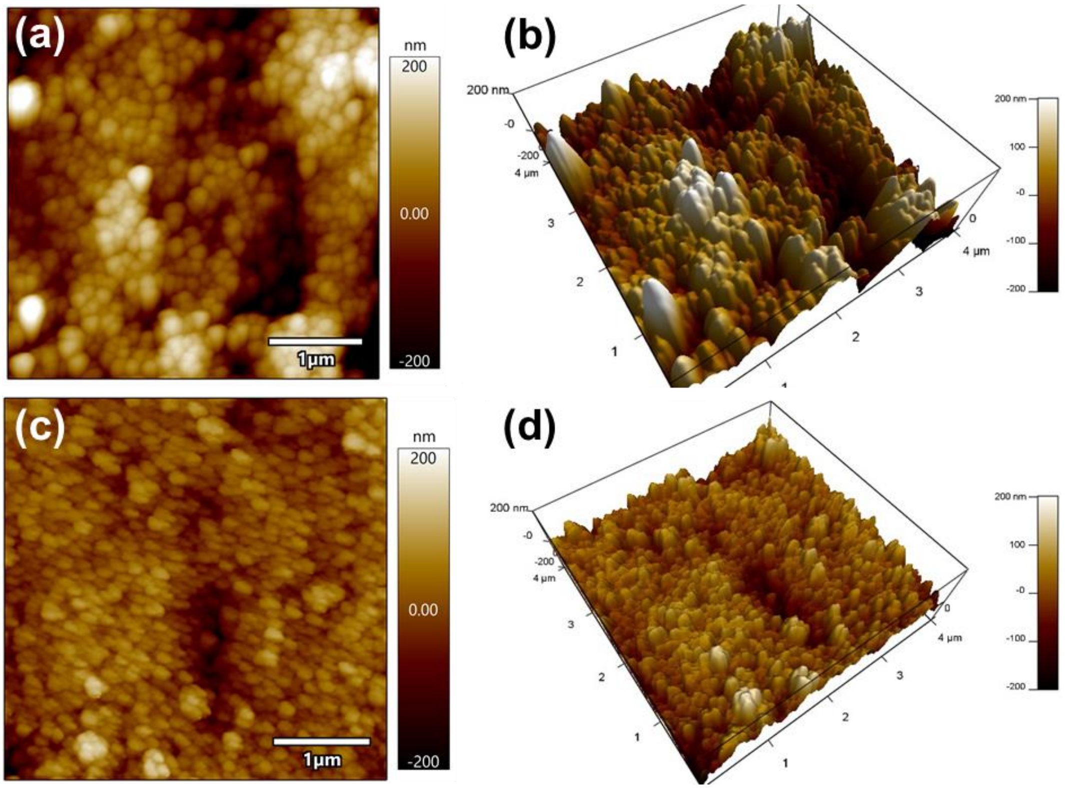

Figure S2. AFM images of (a, b) Cu catalyst and (c, d) F-Cu catalyst.

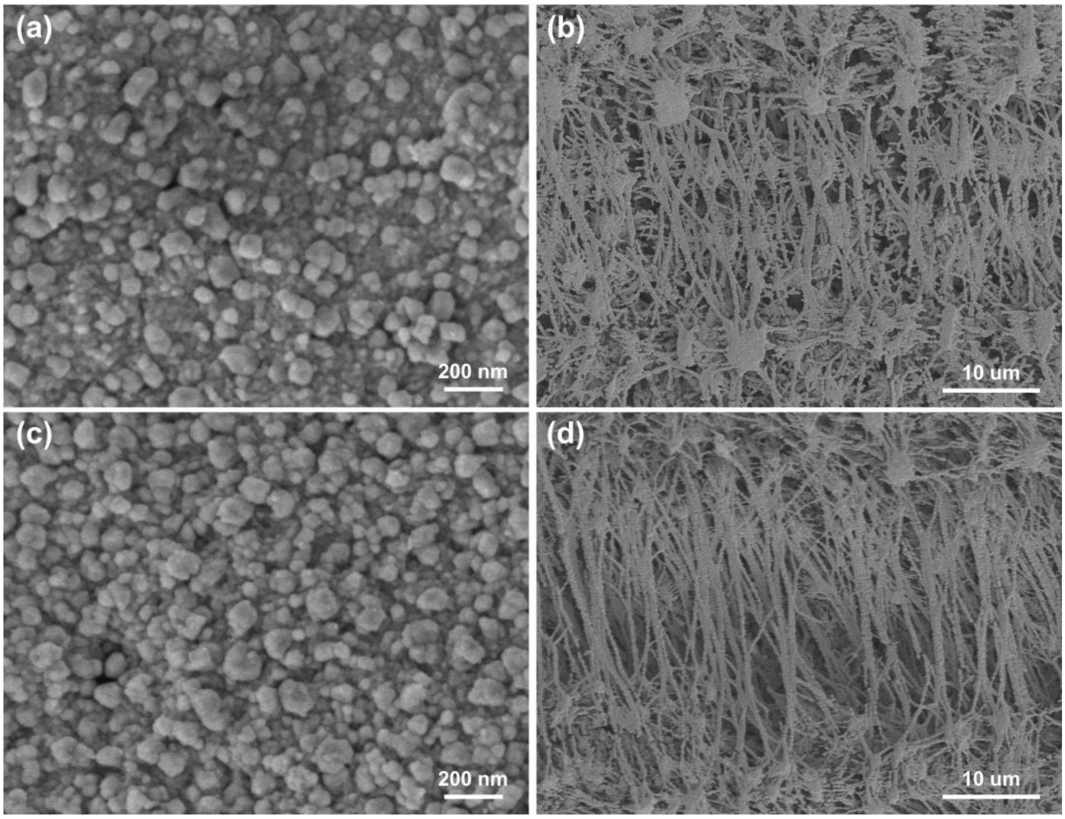

Figure S3. SEM images of (a, b) F-Cu-100 W catalyst and (c, d) F-Cu-150 W catalyst.

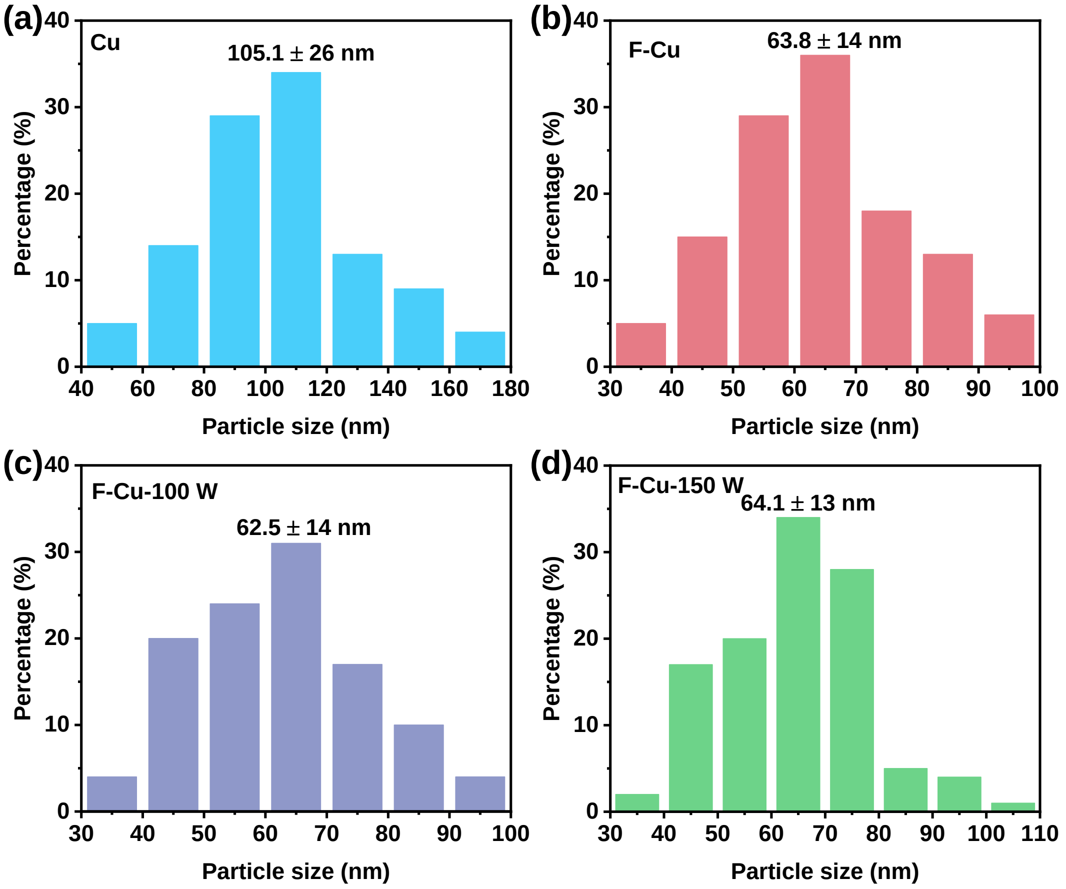

Figure S4. Size distributions of particles in (a) Cu catalyst, (b) F-Cu-200 W catalyst, (c) F-Cu-100 W catalyst, and (d) F-Cu-150 W catalyst.

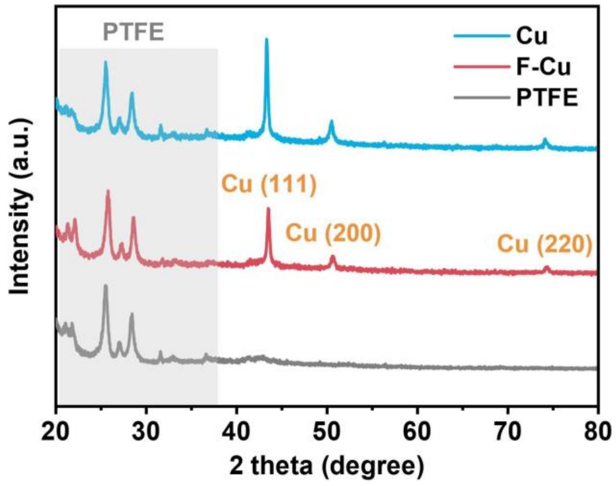

Figure S5. XRD patterns of Cu catalyst, F-Cu catalyst, and PTFE substrate.

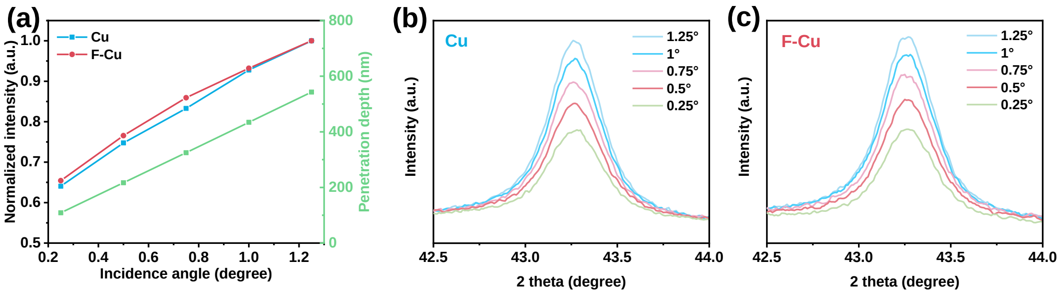

Figure S6. (a) XRD peak intensity of Cu (111) collected at incidence angles varying from 0.25° to 1.25° and normalized by the intensity measured at an incidence angle of 1.25°. Incidence-angle dependent XRD patterns of (b) Cu and (c) F-Cu collected at incidence angles varying from 0.25° to 1.25°.

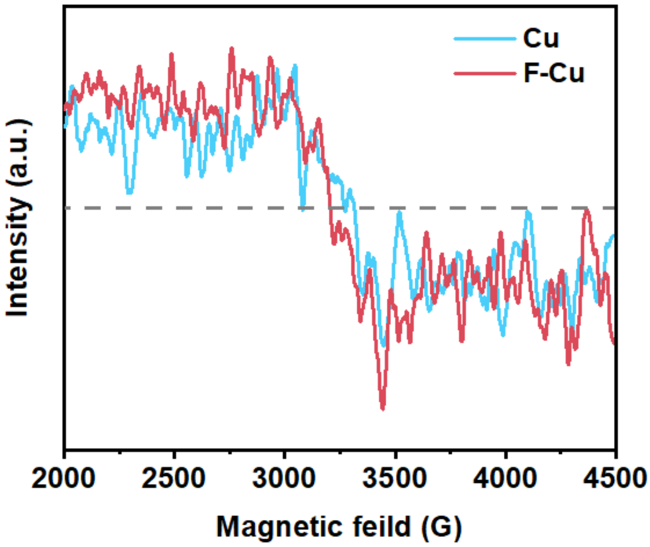

Figure S7. EPR spectra of Cu and F-Cu catalysts.

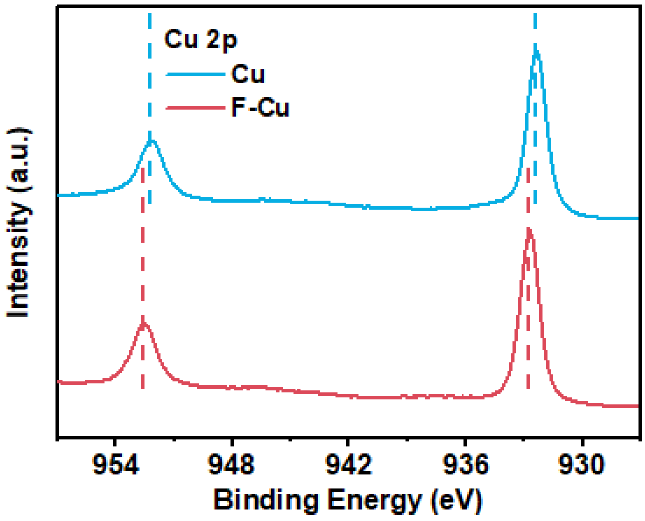

Figure S8. Cu 2p XPS spectra of Cu and F-Cu catalysts.

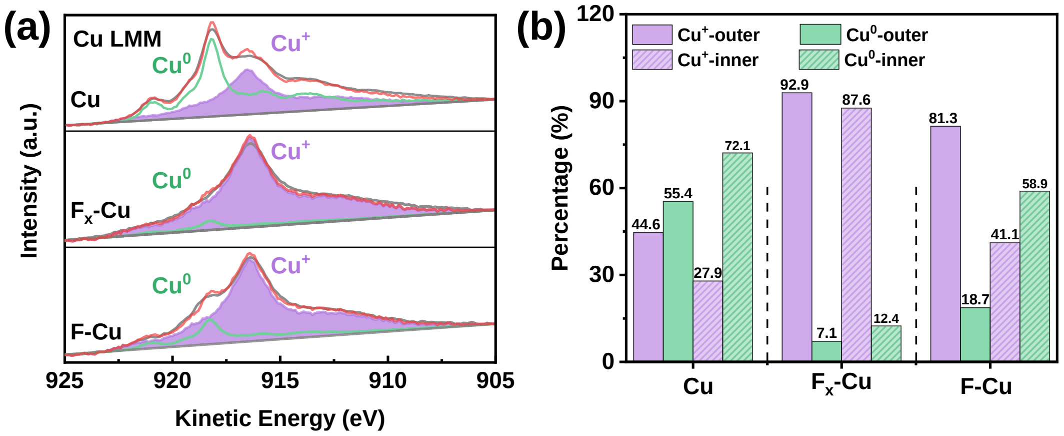

Figure S9. (a) Cu LMM Auger spectra of Cu catalyst, F_x-Cu pre-catalyst, and F-Cu catalyst. (b) The Cu^+ and Cu^0 content of Cu catalyst, F_x-Cu pre-catalyst, and F-Cu catalyst before and after slight surface etching with Ar^+ beam.

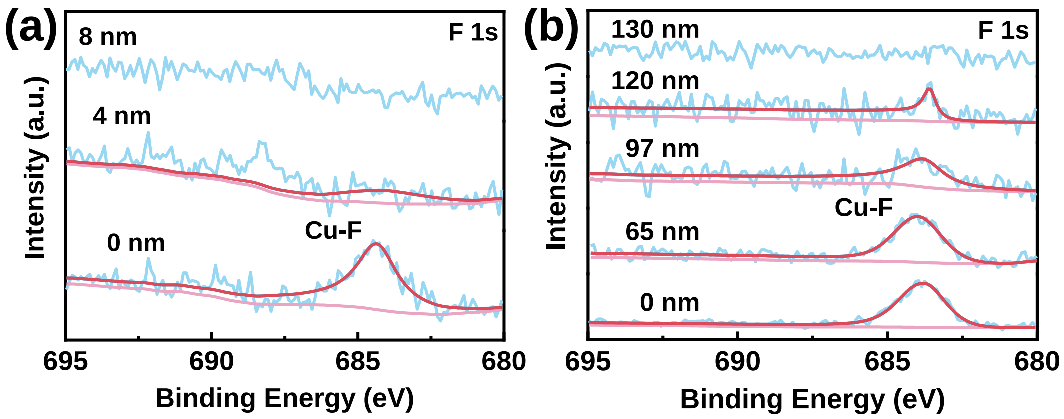

Figure S10. F 1s XPS spectra for (a) F_x-Cu-100 and (b) F_x-Cu-150 with respect to different Ar^+ beam etching depths. The etching depths are estimated values based on theoretical parameters. These numbers of 100, 150 and 200 represent the different powers in the plasma process. These samples were directly fluorinated by CF_4 plasma using copper foil as the precursor.

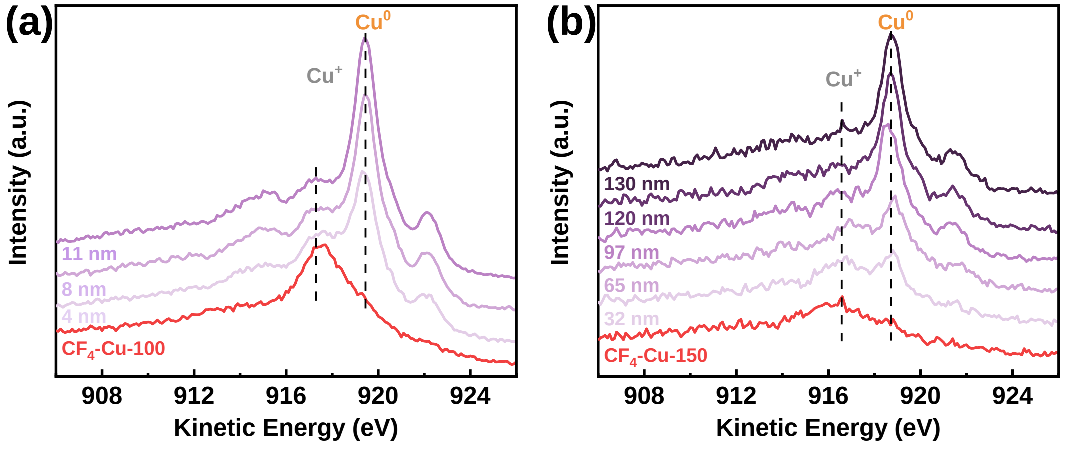

Figure S11. Cu LMM Auger spectra of (a) F_x-Cu-100 and (b) F_x-Cu-150 with respect to different Ar^+ beam etching depths. The etching depths are estimated values based on theoretical parameters.

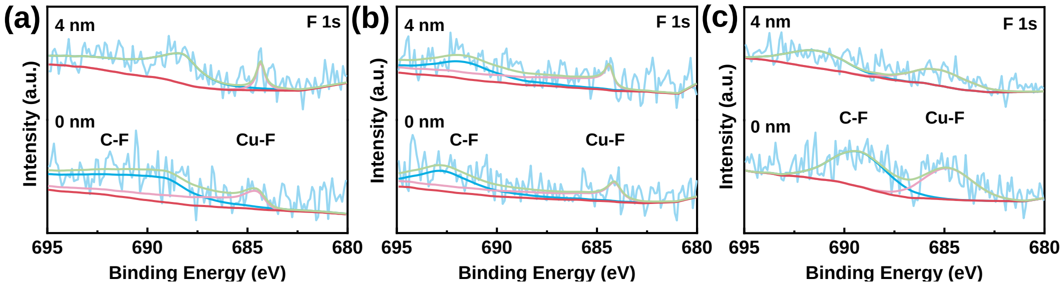

Figure S12. F 1s XPS spectra for (a) F-Cu-100, (b) F-Cu-150, and (c) F-Cu-200 with respect to different Ar^+ beam etching depths. These numbers of 100, 150 and 200 represent the different powers in the plasma process. These samples were directly fluorinated by CF_4 plasma using copper foil as the precursor.

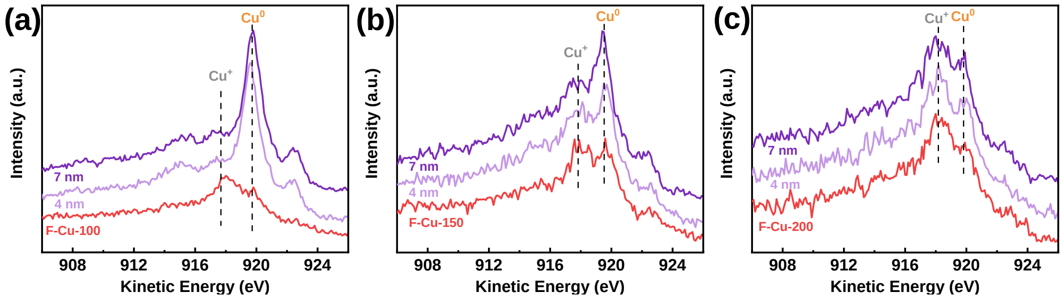

Figure S13. Cu LMM Auger spectra of (a) F-Cu-100, (b) F-Cu-150, and (c) F-Cu-200 with respect to different Ar^+ beam etching depths. The etching depths are estimated values based on theoretical parameters.

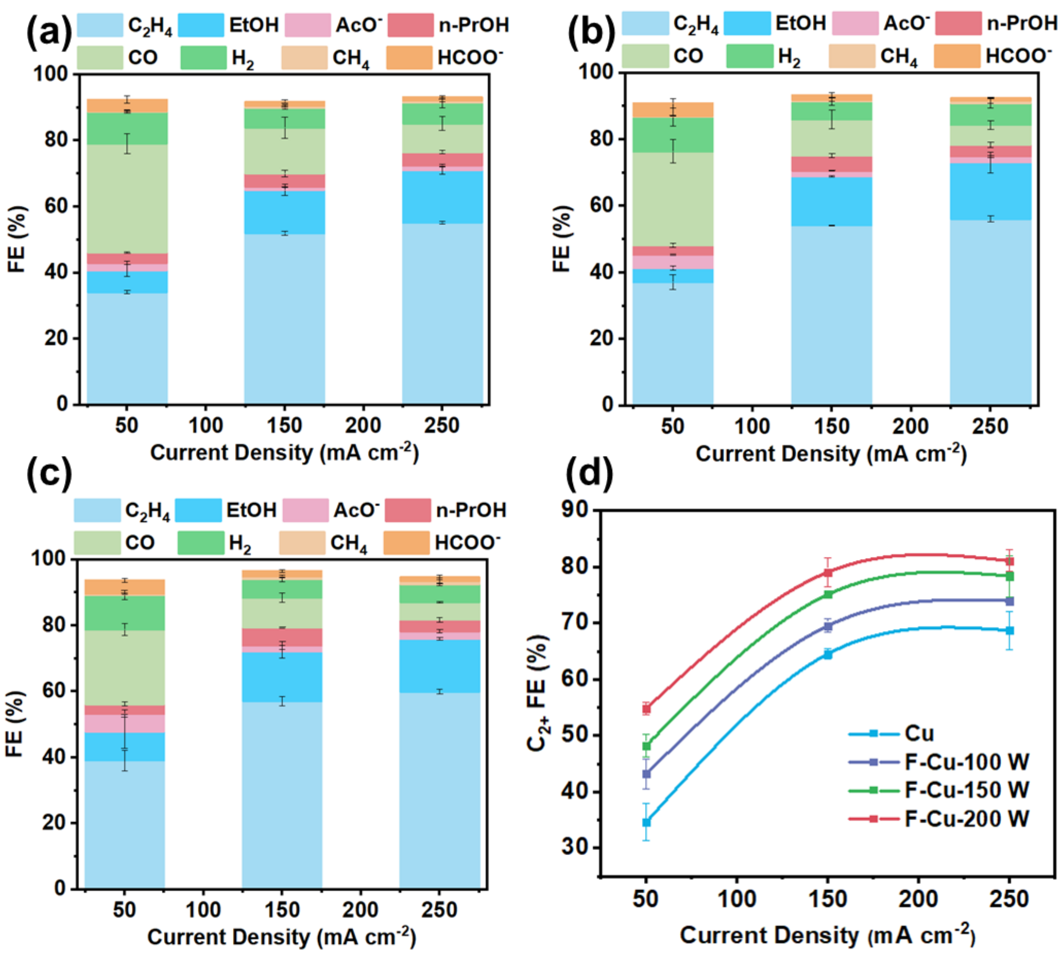

Figure S14. FE values of different products on F-Cu catalyst with different power of (a) 100 W, (b) 150 W and (c) 200 W. (d) C_{2+} FE values from (a), (b), and (c).

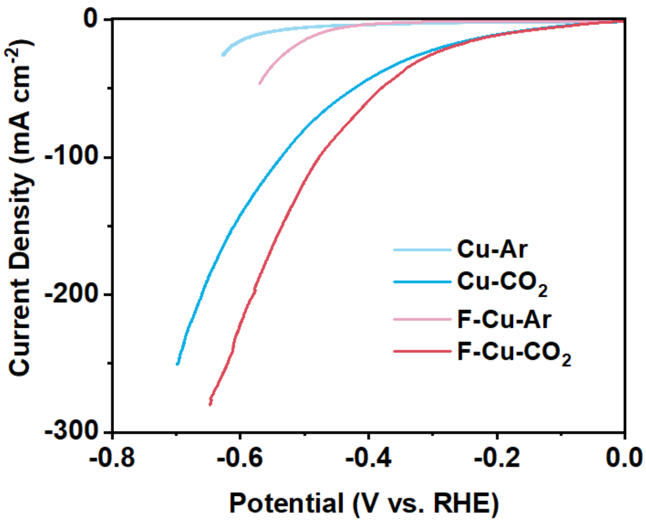

Figure S15. LSV curves in Ar and CO_2 environments at a scan rate of 50 mV s^{-1} over F-Cu catalyst and Cu catalyst in 1.0 M KOH.

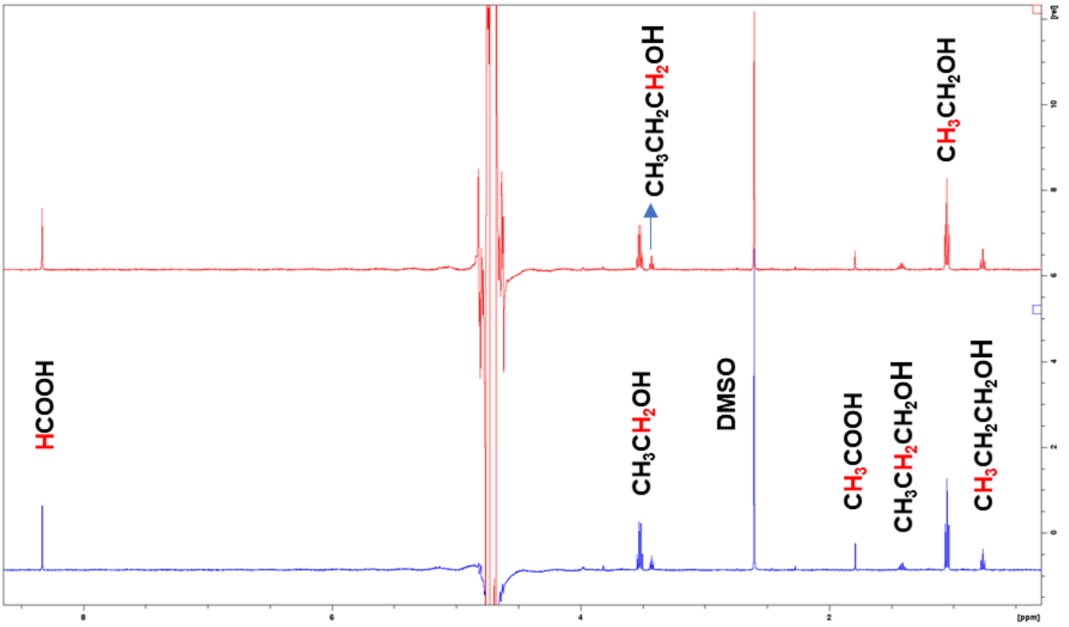

Figure S16. Representative nuclear magnetic resonance (NMR) spectra of the liquid products for F-Cu catalyst (red) and Cu catalyst (blue) at a current density of 100 mA cm^{-2}.

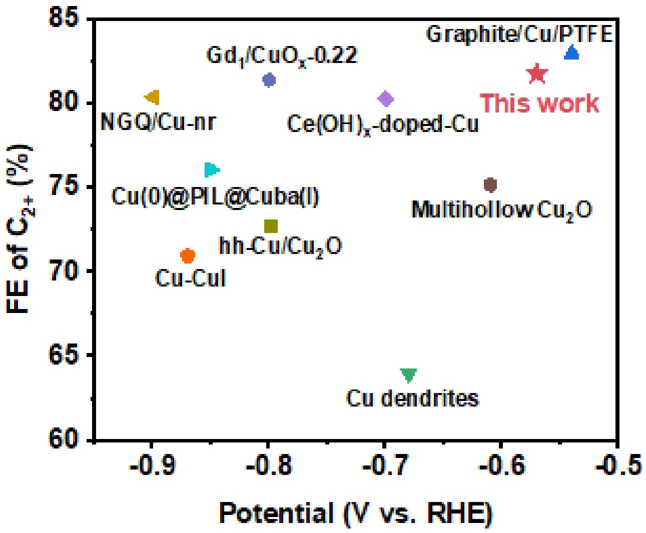

Figure S17. Comparison of electrochemical CO_2RR performance on F-Cu catalyst with reported Cu-based catalysts in the flow cell.

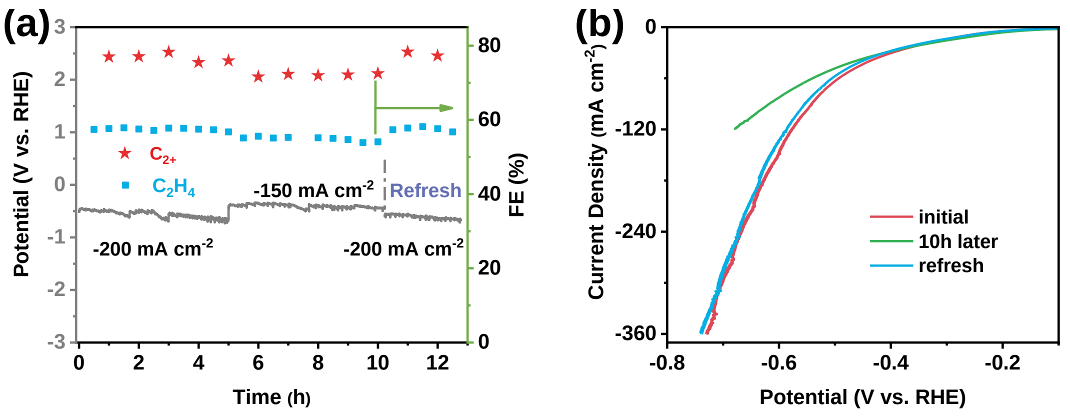

Figure S18. (a) Stability test of the F-Cu catalyst at a current density of 200 mA cm^{-2} and 150 mA cm^{-2}. (b) LSV curves of the F-Cu catalyst before and after the 10 h stability test as well as the LSV curve after the consequent electrolyte refresh.

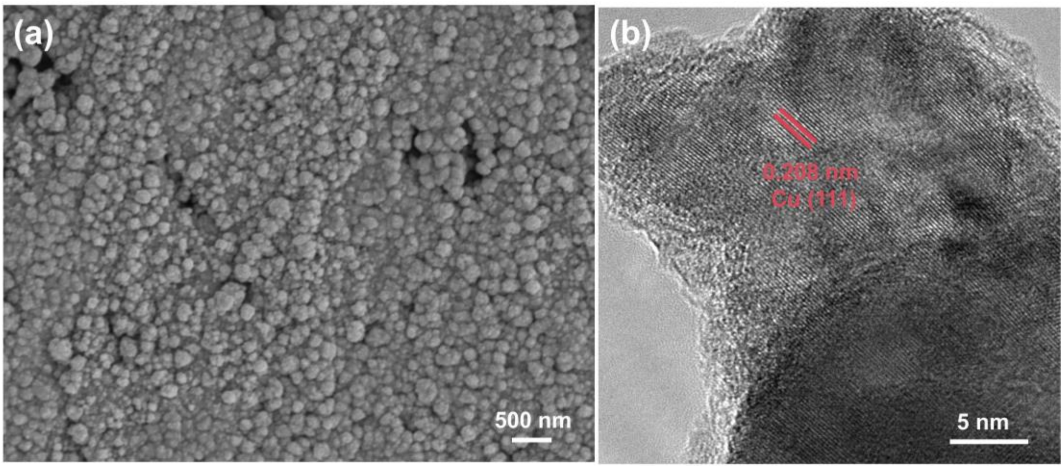

Figure S19. (a) SEM image and HRTEM image of F-Cu catalyst after 12h electrochemical CO_2RR.

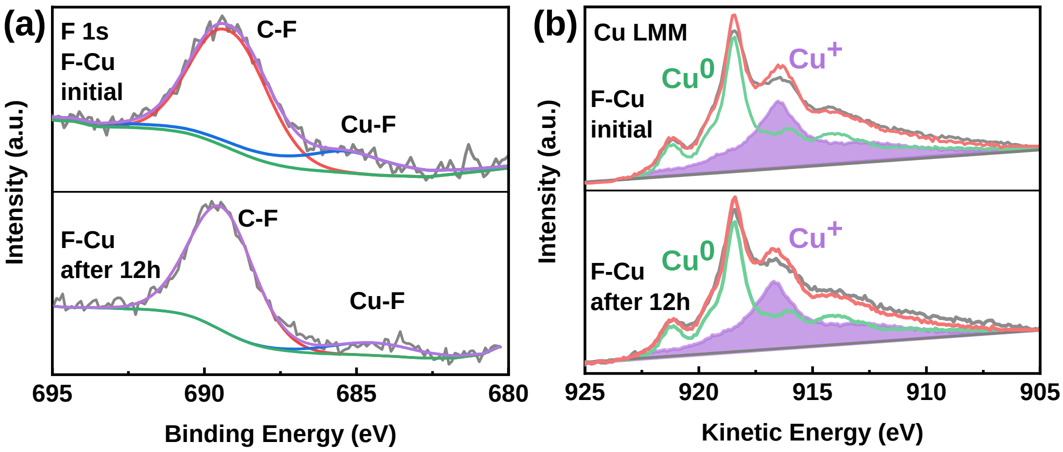

Figure S20. (a) F 1s XPS spectra and (b) Cu LMM Auger spectra of F-Cu catalyst before and after 12 h electrochemical CO_2RR.

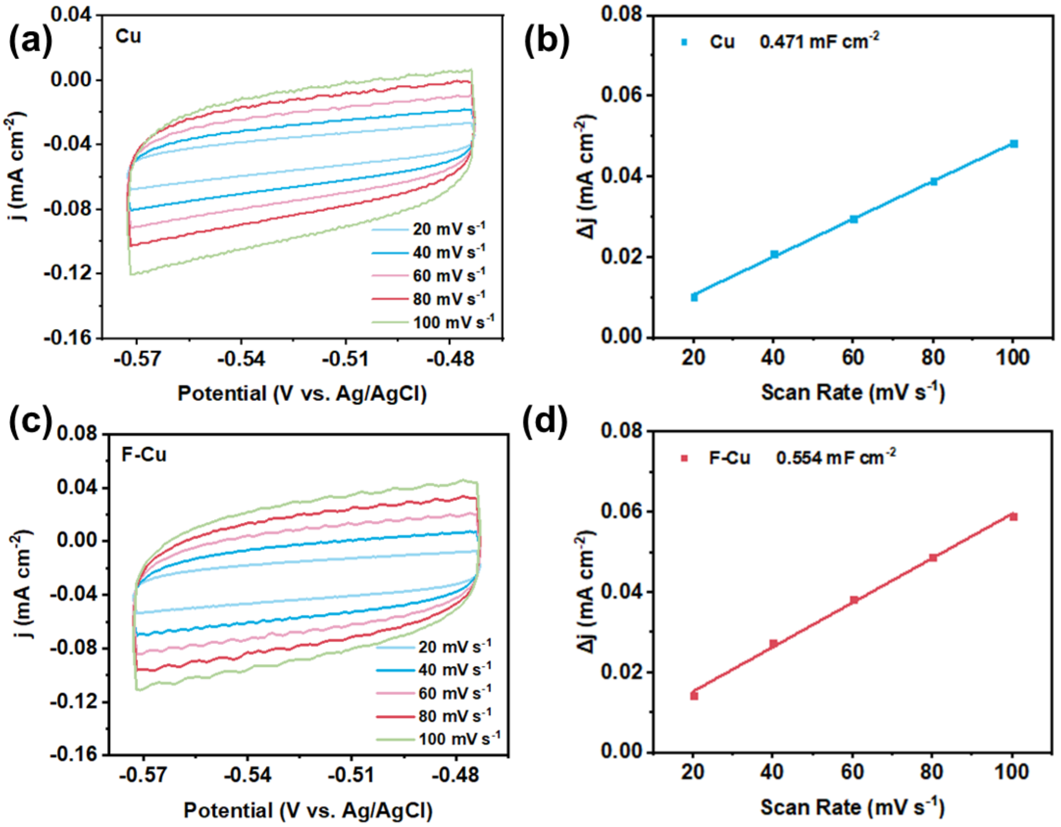

Figure S21. (a) The cyclic voltammetry and (b) double-layer capacitance curves of the Cu catalyst. (c) The cyclic voltammetry and (d) double layer capacitance curve of the F-Cu catalyst. Generally, ECSA = $R_f \times S$, where R_f was calculated from the ratio of double-layer capacitance (C_{dl}) for the working electrode and the corresponding smooth polycrystalline Cu electrode (29 μF cm^{-2}) and S stands for the geometric area of the electrode (in this work, S = 1 cm^2). Therefore, the ECSA are 16.24 and 19.1 cm^2 for the Cu and F-Cu catalysts, respectively.

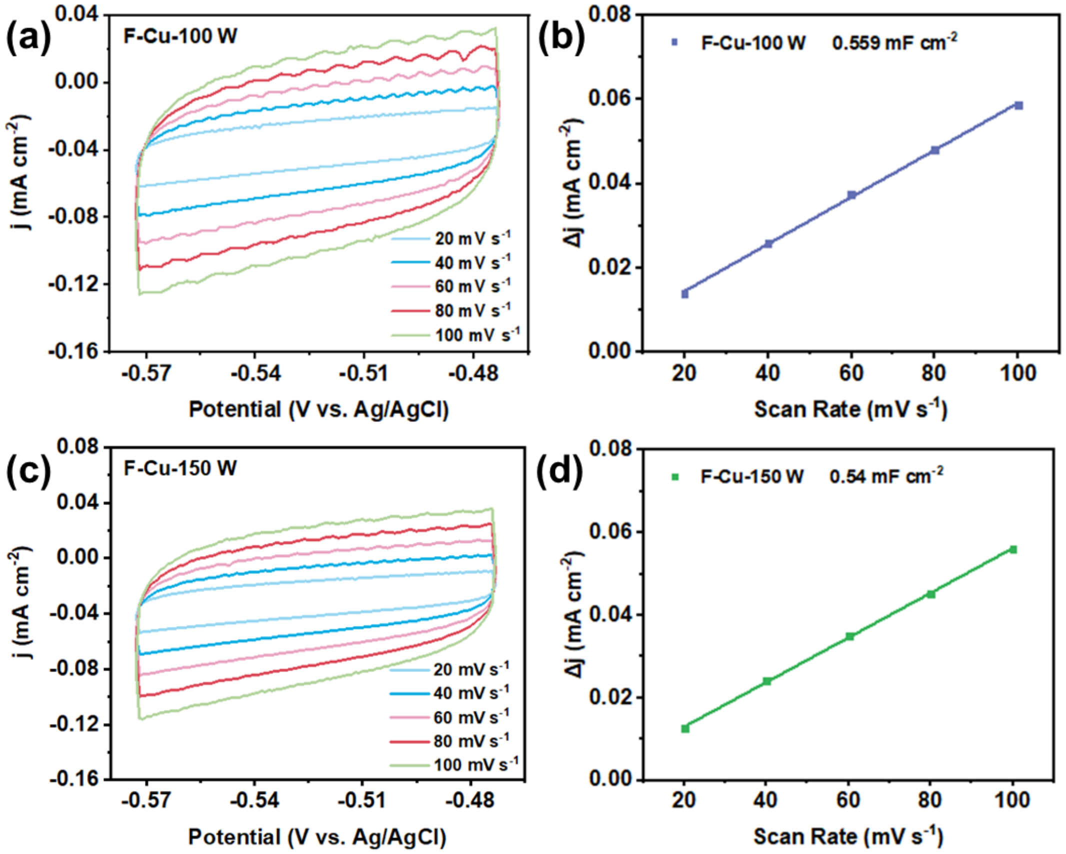

Figure S22. (a) The cyclic voltammetry and (b) double layer capacitance curves of the F-Cu-100 W catalyst. (c) The cyclic voltammetry and (d) double layer capacitance curve of the F-Cu-150 W catalyst.

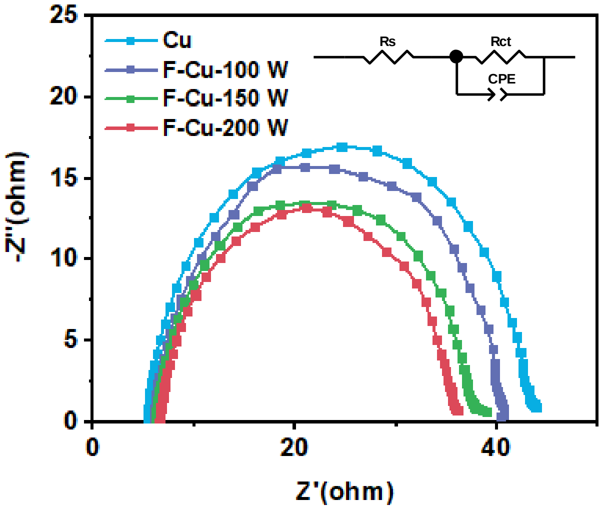

Figure S23. Nyquist plots of Cu catalyst and F-Cu catalyst in 1.0 M KOH at −0.2 V vs. RHE.

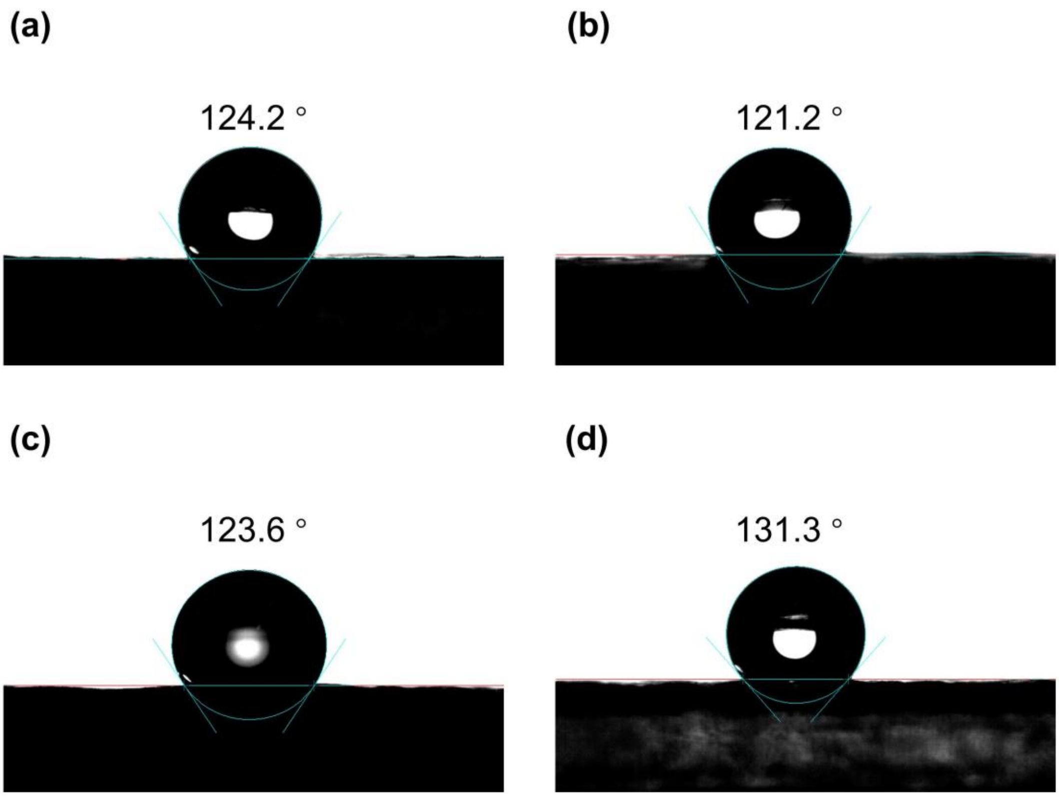

Figure S24. Air/water contact angle of the (a) Cu catalyst, (b) F-Cu-100 W catalyst, (c) F-Cu-150 W catalyst, and (d) F-Cu-200 W catalyst.

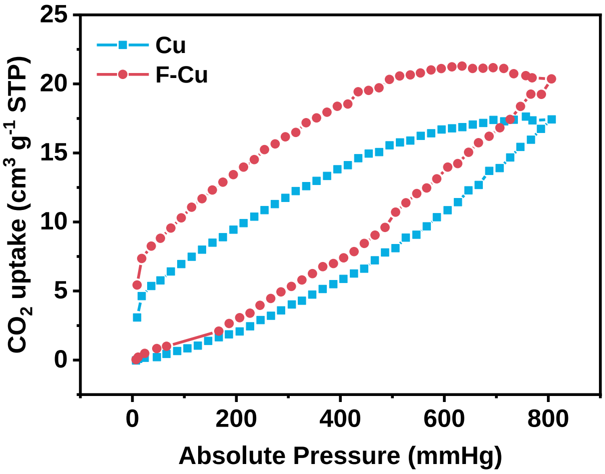

Figure S25. CO_2 sorption isotherms at 273 K of Cu and F-Cu catalysts.

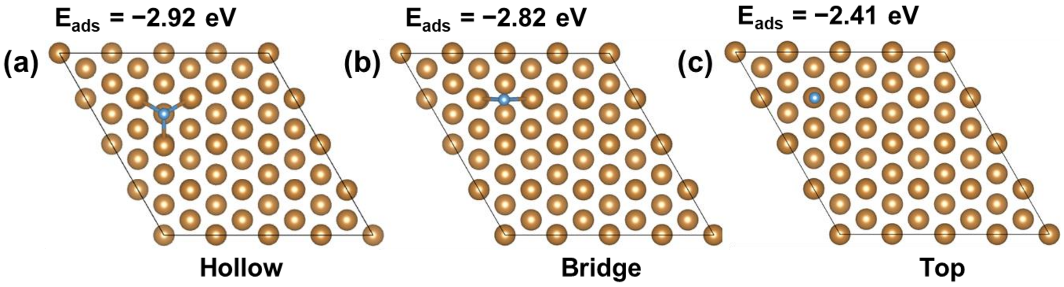

Figure S26. The DFT models of a Cu (111) surface with fluorine adsorption at

various locations and the corresponding adsorption energy.

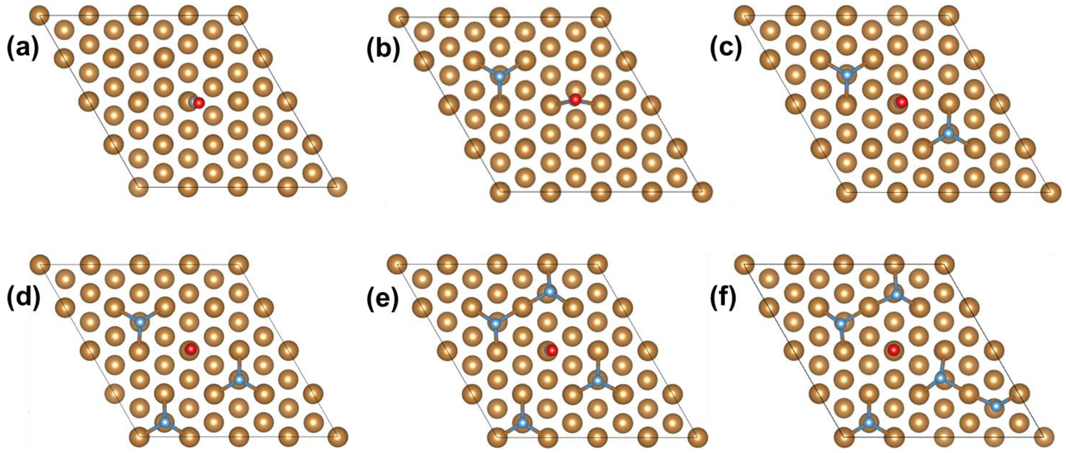

Figure S27. The DFT models of *CO adsorption on different F-Cu surfaces with various concentrations of fluorine.

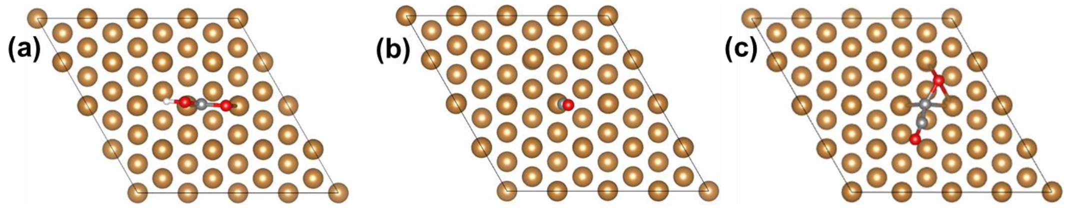

Figure S28. Optimized structures of (a) *COOH, (b) *CO, and (c) *OCCO on Cu (111) surfaces.

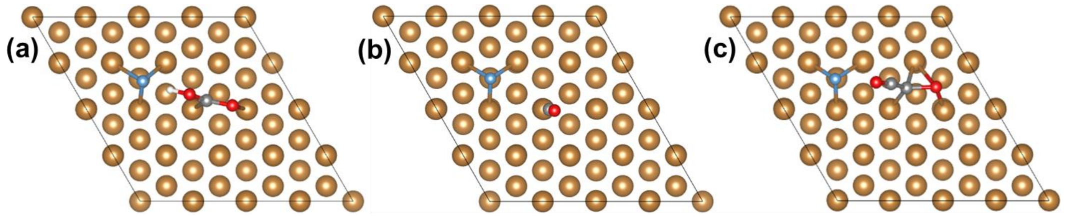

Figure S29. Optimized structures of (a) *COOH, (b) *CO, and (c) *OCCO on F-Cu (111) surfaces.

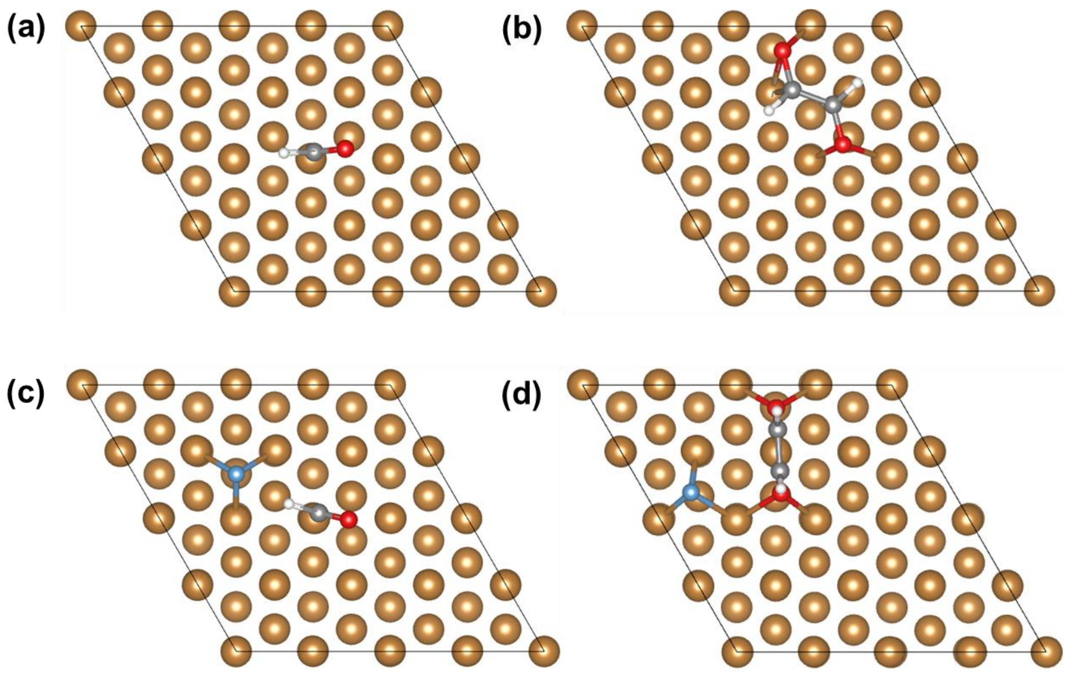

Figure S30. Optimized structures of (a) *CHO on Cu (111) surfaces, (b) *OCHCHO* on Cu (111) surfaces, (c) *CHO on F-Cu (111) surfaces, and (d) *OCHCHO* on F-Cu (111) surfaces.

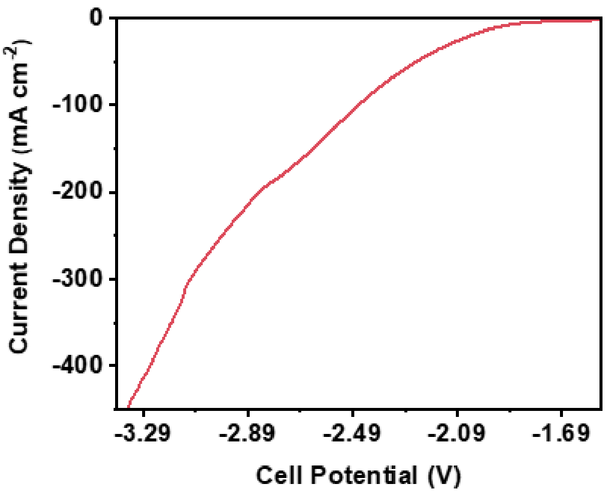

Figure S31. LSV curve at a scan rate of 50 mV s^{-1} over F-Cu catalyst in the MEA system.

Table S1. Comparison of Electrocatalytic performances for CO_2 to C_{2+} products on F-Cu catalyst in a flow cell with reported values on Cu-based catalysts.

Catalyst	Cell type	Electrolyte	E (V)	FE of C_{2+} (%)	Ref.
F-Cu	Flow cell	1 M KOH	−0.57	81.8	This work

F-Cu	MEA	1 M KOH	−3.06*	81.6	This work
Gd_1/CuO_x-0.22	Flow cell	2 M KOH	−0.80	81.4	[7]
AgI-CuO	Flow cell	1 M $KHCO_3$	−1.05*	63.2	[8]
OD-Cu-III	Flow cell	1 M $KHCO_3$	-	74.9	[9]
Graphite/Cu/PTFE	Flow cell	10 M KOH	−0.54	83	[10]
Cu dendrites	Flow cell	1 M KOH	−0.68	64	[11]
$Ce(OH)_x$-doped-Cu	Flow cell	1 M KOH	−0.7	80.3	[12]
NGQ/Cu-nr	Flow cell	1 M KOH	−0.9	80.4	[13]
Cu(0)@PIL@Cuba(I)	Flow cell	1 M KOH	−0.85	76.1	[14]
Multihollow Cu_2O	Flow cell	2 M KOH	−0.61	75.2	[15]
hh-Cu/Cu_2O	Flow cell	1 M NaOH	−0.8	72.8	[16]
Cu_3N_x	Flow cell	1 M KOH	−1.15*	81.7	[17]
Cu-CuI	Flow cell	1 M KOH	−0.87	71	[18]
CuO-CeO_2	Flow cell	1 M KOH	−1.12*	75.2	[19]
N_2SN-Ag-Cu	MEA	0.1 M	~−4.5*	80	[20]

		$KHCO_3$			
Cu/PTFE	MEA	0.1 M $KHCO_3$	−3.9*	~80	[21]
Cu/Fe-N-Cs-GDE	MEA	0.50 M KOH	−3.38*	89.3	[22]

The potential (E) in the flow cell is compared with that of the reversible hydrogen electrode (RHE). *Represents the potential without iR correction.

Table S2. Comparison of Electrocatalytic performances for CO_2 to C_{2+} products on F-Cu catalyst with reported values on Cu-based catalysts modified by nonmetallic heteroatoms.

Catalyst	Cell type	Electrolyte	E (V)	FE of C_{2+} (%)	Ref.
F-Cu	Flow cell	1 M KOH	−0.57	81.8	This work
F-Cu	MEA	1 M KOH	−3.06*	81.6	This work
K-F-Cu	Flow cell	1 M KOH	−0.53	75.3	[23]
Cu(B)	H-cell	0.1 M KCl	−1.1*	79	[24]
F-Cu	Flow cell	0.75 M KOH	−0.89	80	[25]
Cu_KI	H-cell	0.1 M $KHCO_3$	−1.09	72.6	[26]
Cl-doped Cu	H-cell	0.1 M $KHCO_3$	−1*	53.8	[27]
N-Cu	Flow cell	1 M KOH	−1.15	73.7	[28]
I-modified Cu	H-cell	0.50 M KOH	−0.9	80	[29]

The potential (E) in the flow cell and H-cell is compared with that of the reversible hydrogen electrode (RHE). * Represents the potential without iR correction.

References

[1] S. Nitopi, E. Bertheussen, S. B. Scott, X. Liu, A. K. Engstfeld, S. Horch, B. Seger, I. E. L. Stephens, K. Chan, C. Hahn, J. K. Nørskov, T. F. Jaramillo, I. Chorkendorff, *Chem. Rev.* **2019**, *119*, 7610-7672.

[2] G. Kresse, D. Joubert, *Phys. Rev. B* **1999**, *59*, 1758-1775.

[3] J. P. Perdew, K. Burke, M. Ernzerhof, *Phys. Rev. Lett.* **1996**, *77*, 3865-3868.

[4] S. Grimme, J. Antony, S. Ehrlich, H. Krieg, *J. Chem. Phys.* **2010**, *132*, 154104.

[5] J. K. Nørskov, J. Rossmeisl, A. Logadottir, L. Lindqvist, J. R. Kitchin, T. Bligaard, H. Jónsson, *J. Phys. Chem. B* **2004**, *108*, 17886-17892.

[6] V. Wang, N. Xu, J.-C. Liu, G. Tang, W.-T. Geng, *Comput. Phys. Commun.* **2021**, *267*, 108033.

[7] J. Feng, L. Wu, S. Liu, L. Xu, X. Song, L. Zhang, Q. Zhu, X. Kang, X. Sun, B. Han, *J. Am. Chem. Soc.* **2023**, *145*, 9857-9866.

[8] R. Yang, J. Duan, P. Dong, Q. Wen, M. Wu, Y. Liu, Y. Liu, H. Li, T. Zhai, *Angew. Chem. Int. Ed.* **2022**, *61*, e202116706.

[9] Z.-Z. Wu, X.-L. Zhang, Z.-Z. Niu, F.-Y. Gao, P.-P. Yang, L.-P. Chi, L. Shi, W.-S. Wei, R. Liu, Z. Chen, S. Hu, X. Zheng, M.-R. Gao, *J. Am. Chem. Soc.* **2022**, *144*, 259-269.

[10] C.-T. Dinh, T. Burdyny, M. G. Kibria, A. Seifitokaldani, C. M. Gabardo, F. P. García de Arquer, A. Kiani, J. P. Edwards, P. De Luna, O. S. Bushuyev, C. Zou, R. Quintero-Bermudez, Y. Pang, D. Sinton, E. H. Sargent, *Science* **2018**, *360*, 783-787.

[11] Z.-Z. Niu, F.-Y. Gao, X.-L. Zhang, P.-P. Yang, R. Liu, L.-P. Chi, Z.-Z. Wu, S. Qin, X. Yu, M.-R. Gao, *J. Am. Chem. Soc.* **2021**, *143*, 8011-8021.

[12] M. Luo, Z. Wang, Y. C. Li, J. Li, F. Li, Y. Lum, D.-H. Nam, B. Chen, J. Wicks, A. Xu, T. Zhuang, W. R. Leow, X. Wang, C.-T. Dinh, Y. Wang, Y. Wang, D. Sinton, E. H. Sargent, *Nat. Commun.* **2019**, *10*, 5814.

[13] C. Chen, X. Yan, S. Liu, Y. Wu, Q. Wan, X. Sun, Q. Zhu, H. Liu, J. Ma, L. Zheng, H. Wu, B. Han, *Angew. Chem. Int. Ed.* **2020**, *59*, 16459-16464.

[14] G.-Y. Duan, X.-Q. Li, G.-R. Ding, L.-J. Han, B.-H. Xu, S.-J. Zhang, *Angew. Chem. Int. Ed.* **2022**, *61*, e202110657.

[15] P.-P. Yang, X.-L. Zhang, F.-Y. Gao, Y.-R. Zheng, Z.-Z. Niu, X. Yu, R. Liu, Z.-Z. Wu, S. Qin, L.-P. Chi, Y. Duan, T. Ma, X.-S. Zheng, J.-F. Zhu, H.-J. Wang, M.-R. Gao, S.-H. Yu, *J. Am. Chem. Soc.* **2020**, *142*, 6400-6408.

[16] J. Li, K. Xu, F. Liu, Y. Li, Y. Hu, X. Chen, H. Wang, W. Xu, Y. Ni, G. Ding, T. Zhao, M. Yu, W. Xie, F. Cheng, *Adv. Mater.* **2023**, *35*, 2301127.

[17] C. Peng, G. Luo, Z. Xu, S. Yan, J. Zhang, M. Chen, L. Qian, W. Wei, Q. Han, G. Zheng, *Adv. Mater.* **2021**, *33*, 2103150.

[18] H. Li, T. Liu, P. Wei, L. Lin, D. Gao, G. Wang, X. Bao, *Angew. Chem. Int. Ed.* **2021**, *60*, 14329-14333.

[19] X. Yan, C. Chen, Y. Wu, S. Liu, Y. Chen, R. Feng, J. Zhang, B. Han, *Chem. Sci.* **2021**, *12*, 6638-6645.

[20] G. W. K. Moore, S. E. L. Howell, M. Brady, X. Xu, K. McNeil, *Nat. Commun.* **2021**, *12*, 1.

[21] C. M. Gabardo, C. P. O'Brien, J. P. Edwards, C. McCallum, Y. Xu, C.-T. Dinh, J.

Li, E. H. Sargent, D. Sinton, *Joule* **2019**, *3*, 2777-2791.
[22]T. Zhang, J. C. Bui, Z. Li, A. T. Bell, A. Z. Weber, J. Wu, *Nat. Catal.* **2022**, *5*, 202-211.
[23]C. Peng, S. Yang, G. Luo, S. Yan, M. Shakouri, J. Zhang, Y. Chen, W. Li, Z. Wang, T.-K. Sham, G. Zheng, *Adv. Mater.* **2022**, *34*, 2204476.
[24]Y. Zhou, F. Che, M. Liu, C. Zou, Z. Liang, P. De Luna, H. Yuan, J. Li, Z. Wang, H. Xie, H. Li, P. Chen, E. Bladt, R. Quintero-Bermudez, T.-K. Sham, S. Bals, J. Hofkens, D. Sinton, G. Chen, E. H. Sargent, *Nat. Chem.* **2018**, *10*, 974-980.
[25]W. Ma, S. Xie, T. Liu, Q. Fan, J. Ye, F. Sun, Z. Jiang, Q. Zhang, J. Cheng, Y. Wang, *Nat. Catal.* **2020**, *3*, 478-487.
[26]T. Kim, G. T. R. Palmore, *Nat. Commun.* **2020**, *11*, 3622.
[27]M. Li, Y. Ma, J. Chen, R. Lawrence, W. Luo, M. Sacchi, W. Jiang, J. Yang, *Angew. Chem. Int. Ed.* **2021**, *60*, 11487-11493.
[28]M. Zheng, P. Wang, X. Zhi, K. Yang, Y. Jiao, J. Duan, Y. Zheng, S.-Z. Qiao, *J. Am. Chem. Soc.* **2022**, *144*, 14936-14944.
[29]D. Gao, I. Sinev, F. Scholten, R. M. Arán-Ais, N. J. Divins, K. Kvashnina, J. Timoshenko, B. R. Cuenya, *Angew. Chem. Int. Ed.* **2019**, *58*, 17047-17053.
